# Nucleophile responsive charge-reversing polycations for pDNA transfection†

**DOI:** 10.1039/d3py00075c

**Published:** 2023-03-09

**Authors:** Reece W. Lewis, Aswin Muralidharan, Benjamin Klemm, Pouyan E. Boukany, Rienk Eelkema

**Affiliations:** a Department of Chemical Engineering, Delft University of Technology 2629 HZ Delft The Netherlands R.W.Lewis@tudelft.nl R.Eelkema@tudelft.nl; b Department of Bionanoscience, Delft University of Technology 2629 HZ Delft The Netherlands; c Kavli Institute of Nanoscience, Delft University of Technology 2629 HZ Delft The Netherlands

## Abstract

Polycationic carriers promise low cost and scalable gene therapy treatments, however inefficient intracellular unpacking of the genetic cargo has limited transfection efficiency. Charge-reversing polycations, which transition from cationic to neutral or negative charge, can offer targeted intracellular DNA release. We describe a new class of charge-reversing polycation which undergoes a cationic-to-neutral conversion by a reaction with cellular nucleophiles. The deionization reaction is relatively slow with primary amines, and much faster with thiols. In mammalian cells, the intracellular environment has elevated concentrations of amino acids (∼10×) and the thiol glutathione (∼1000×). We propose this allows for decationization of the polymeric carrier slowly in the extracellular space and then rapidly in the intracellular milleu for DNA release. We demonstrate that in a lipopolyplex formulation this leads to both improved transfection and reduced cytotoxicity when compared to a non-responsive polycationic control.

## Abstract 2

A new class of charge-reversing polycation is studied for transfection of mammalian cells. In a lipopolyplex formulation, we observed improved transfection and reduced toxicity compared to a permanently cationic control.

## Graphical abstract

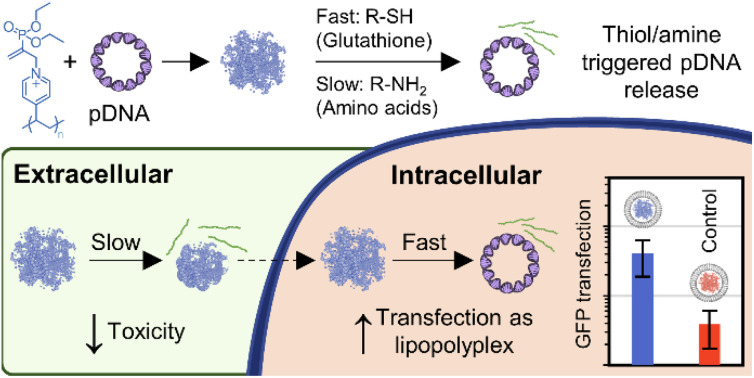

## Introduction

Gene therapy holds great promise as a treatment for genetic diseases such as cancer, cardiovascular disease, cystic fibrosis and arthritis.^1^ These treatments require nucleic acid cargo to reach the intracellular milieu, and in the case of plasmid DNA (pDNA), the nucleus. A carrier or vector is used to protect the cargo from serum nucleases and facilitate uptake into the cell.^2^ Viral vectors are generally the most efficacious in terms of transfection, however they suffer from high cost, specialised production and safety concerns around insertional mutagenesis and immune responses.^3^ This has promoted the study of nonviral vectors such as cationic polymers, which promise low pathogenicity and cheap scalable production.^4,5^

Cationic polymers can electrostatically bind to negatively charged nucleic acids, resulting in the formation of nanoparticle complexes. These are typically termed polyplexes when the polycation is a homopolymer, while block copolymers with a neutral, water-soluble block yield polyplex micelles, which have a protective corona.^2,6^ This corona typically conveys polyplex micelles with improved extracellular stability, however it can also reduce cellular internalisation, leading to poor transfection.^7^ An alternative is to coat polyplexes with lipids to form lipopolyplexes, combining favourable characteristics from polyplexes (facilitated endosomal release and nucleus entry) and lipid complexes (improved cytotoxicity and cellular uptake).^8–13^

Polyplex performance can be tuned by varying the ratio of positive charges on the polycation to negative charges on the nucleic acid.^5^ An excess of cationic charge is typically used to achieve sufficient DNA packaging and cellular entry, however this can limit complex dissociation once inside the cell, resulting in poor transcription efficiency.^4,14,15^ Additionally, highly cationic polyplexes are often cytotoxic, since cationic polymers can damage cell membranes.^2,12^ This has motivated the development of charge-reversing polycations which are able to transition from cationic to neutral or negative charge, either gradually through hydrolysis at physiological pH,^15–20^ or in response to intracellular signals such as reactive oxygen species (ROS),^7,21^ and esterases.^22,23^ For example, Zhu *et al.* reported reduced cytotoxicity and improved *in vitro* luciferase expression for their ROS responsive polysulfonium polyplexes when compared to permanently cationic controls.^21^ We note, however, that many of these systems are either relatively non-specific for the intracellular environment (hydrolysis), or rely on signals which are mainly elevated inside cancerous cells, limiting scope of application.

**Fig. 1:**
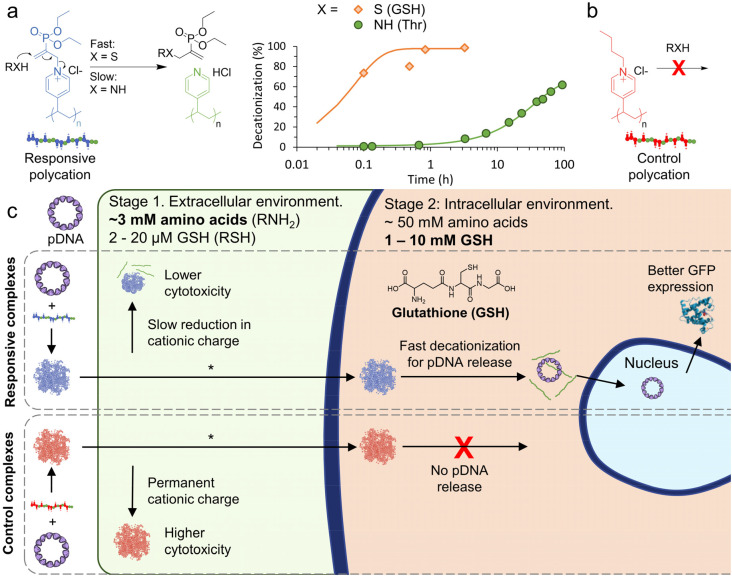
Outline of nucleophile responsive charge-reversing polycation study. (a) General structure of responsive polycation and its charge-reversing reaction with S/N-nucleophiles. Representative ^1^H NMR conversion data for the decationization reaction in phosphate buffer (pH 7.4, 100 mM) at RT ([cationic units] = [nucleophile] = 7 mM), see Fig. S1 and S2† for additional data. Curves are fit with pseudo first-order kinetic model. (b) General structure of non-responsive polycation. (c) Scheme showing proposed two-stage charge reversing process of responsive pDNA complexes in extracellular and intracellular conditions. *In this work cellular transfection is demonstrated by combining the polymer complexes with a lipid.

Thiol/amine nucleophiles (S/N), which can be found in a wide range of biomolecules with typically elevated intracellular concentrations, are until now an unexplored class of charge-reversing signal. We were inspired to investigate nucleophile responsive polycations after our recent work demonstrating controlled (de)cationization of polyvinylpyridine (PVP) copolymers by successive additions of an allyl acetate (ionization), and S/N-nucleophiles (deionization).^24,25^ With an appropriate polyanion, the PVP copolymers (dis)assembled into coacervate core micelles, effectively achieving signal controlled polyanion encapsulation and release.^26^ Importantly, we found that the decationiziation reaction is significantly faster for thiols compared to primary amines (Fig. 1a). We therefore hypothesised that the responsive cationic PVP could be applied as a two-stage charge-reversing polymer within polyplexes.

The first stage would occur extracellularly, where amine moieties on free amino acids (∼3 mM in human blood plasma),^27,28^ could be exploited to slowly reduce cationic charge, leading to reduced extracellular cytotoxicity. Once inside the cell, the second stage can begin where an elevated concentration of amino acids (∼50 mM)^29,30^ and the thiol glutathione (GSH, 1–10 mM in cytosol and 2–20 μM extracellularly),^3,31^ could be used to trigger an accelerated decationization and release of pDNA for transfection (Fig. 1c).

## Methods

### Materials

*N*,*N*-Dimethylacrylamide (DMA, 99%) and 4-vinylpyridine (4VP, 95%) were obtained from Sigma Aldrich and passed through basic alumina prior to use to remove inhibitor. 2-(Dodecylthiocarbonothioylthio)-2-methylpropionic acid (DDMAT, 98%), 4-((((2-carboxyethyl)thio)carbonothioyl)thio)-4-cyanopentanoic acid (CETCPA, 95%, ABCR), 7-diethylamino-3-[*N*-(3-maleimidopropyl)carbamoyl]coumarin (coumarin maleimide, 97%), bovine serum albimum (BSA, ≥96%), butyl bromide (BuBr, 99%), HAMS F-12 no l-glutamine, no phenol red (Bio-techne), l-buthionine sulfoximine (BSO), l-glutathione reduced (GSH, 98%), l-threonine (Thr, ≥98%) were all used as received and purchased from Merck/Sigma Aldrich unless stated otherwise. CDCl_3_ (99.8% D), D_2_O (99.96% D), d_8_-THF (99.5% D) were obtained from Eurisotop. Diethyl(α-acetoxymethyl) vinylphosphonate (DVP) was synthesised according to our previous work.^24^

The lipids 1,2-dioleoyl-*sn-glycero*-3-phosphoethanolamine (DOPE) and 3β-[*N*-(*N*′,*N*′-dimethylaminoethane)-carbamoyl]cholesterol hydrochloride (DC-Chol) were purchased from Avanti Polar Lipids Company. Nutrient Mixture Ham's F-12 (Sigma Aldrich), fetal bovine serum (FBS, Sigma Aldrich) and 0.25% Trypsin-EDTA (Gibco) were used as received. pEGFP-C1 plasmid (4.7 kb) was amplified with competent *E. coli* cells (Invitrogen™ Subcloning Efficiency™ DH5α Competent Cells), transformed using the heat shock method. After transformation, the cells were cultured overnight in LB broth w/Kanamycin and then the plasmids were harvested using the GeneJET plasmid mini prep kit.

### NMR spectroscopy

^1^H NMR and ^13^C NMR spectra were recorded on an Agilent 400-MR DD2 spectrometer (399.7 and 100.5 MHz, respectively).

### Gel permeation chromatography (GPC)

The average molecular weight and dispersity (Đ) of the synthesized polymers was measured using a Shimadzu GPC with DMF LiBr (25 mM) as eluent with a flow rate of 1.0 mL min^−1^ at 50 °C. The system was equipped with a Shimadzu RID-10A refractive index detector, a Shimadzu SPD-20A UV-Vis detector, PLgel guard column (MIXED, 5 μm; 50 mm × 7.5 mm), and Agilent PLGel column (MIXED-C, 5 μm; 300 mm × 7.5 mm), providing an effective molar mass range of 200 to 2 × 10^6^ g mol^−1^. The GPC columns were calibrated with low dispersity PMMA standards (Sigma Aldrich) ranging from 800 to 2.2 × 10^6^ g mol^−1^, and molar masses are reported as PMMA equivalents. A 3rd-order polynomial was used to fit the log Mp *vs.* time calibration curve, which was near linear across the molar mass ranges.

### Dynamic light scattering (DLS)

Hydrodynamic diameters were evaluated using cumulant method on a Malvern Zetasizer Nano ZS employing a 633 nm laser at a back-scattering angle of 173°. Sizes are reported as intensity weighted harmonic mean size (*Z*-average diameter), with each data point from 10 runs of 5 seconds. Zeta potential measurements were conducted in folded capillary zeta cells (Malvern, DTS1070). Each zeta potential data point was from 2 or 3 measurements of a minimum of 10 runs. For pDNA release studies the temperature inside the cell was set to 37 °C (all other studies at 25 °C).

### Transmission election microscopy (TEM)

TEM measurements were performed on a JEOL JEM-1400plus TEM operated at 120 kV. Samples were prepared by placing 3 μL of the solution onto carbon coated copper grids and incubating for 1 minute followed by blotting the excess solution onto filter paper. The samples were then washed (2×) with of milli-q water, followed by removing the excess onto filter paper. For stained samples, uranyl acetate (UA) solution (3 μL, 2% (w/v)) was applied for 30 seconds before removing the excess onto filter paper. Images were analysed using Image J software by manually measuring the length of the longest dimension across 3 images (*n* ≥ 30). Values are reported as mean ± SD.

### Gel electrophoresis

1% agarose (Promega) gels mixed with 0.01% SYBR Safe were prepared in TAE buffer. SYBR Safe can bind to exposed DNA and emit a fluorescence signal. The samples (10 μL) were first mixed with 6× DNA loading dye (1.2 μL, thermofisher) and loaded into wells prepared in the agarose gel. A voltage drop of 100 V was applied for a duration of 45 minutes in a Mupid One Advance electrophoresis system. The gels were then imaged in a gel imager (BIO-Rad Gel Doc XR+) with Image Lab software.

### Polyvinylpyridine (block-co)polymer synthesis

1. DDMAT (97 mg, 0.27 mmol), 4VP (3.36 g, 32 mmol), and ethanol (4.5 mL) were combined in a glass tube sealed with rubber septum. The reaction mixture was deoxygenated by bubbling with argon for 30 minutes and placed into a LED reactor (444 nm, see previous work for set up details).^26^ The reaction was quenched after 11 h irradiation (71% conversion by ^1^H NMR spectroscopy, *M*_n,conv_ = 9.2 kDa) by removing the glass tube from the light source and opening to air. The polymer was then precipitated into hexane and dried in a vacuum oven overnight (50 °C) to 1.84 g yellow solid (1).

The polyvinylpyridine block co-polymer (2) was prepared in a two-step process starting with the synthesis of a water-soluble macro-CTA (pDMA_261_). For this, CETCPA (82.2 mg, 0.27 mmol), DMA (7.93 g, 80 mmol), DSS (21.3 mg, 0.10 mmol, as internal std) and DI water (11.7 mL) were combined in a glass tube sealed with rubber septum. The reaction mixture was deoxygenated by bubbling with argon for 30 minutes and placed into a LED reactor (444 nm). The reaction was quenched after 2 h irradiation (87% conversion by ^1^H NMR spectroscopy, *M*_n,conv_ = 26.2 kDa) by removing the glass tube from the light source and opening to air. The polymer was then purified by dialysis (MWCO 500–1000 Da), followed by freeze-drying to obtain 6.2 g of a light-yellow powder (pDMA_261_).

2. pDMA_261_ (1.86 g, 0.071 mmol), 4VP (1.22 g, 11.6 mmol), DSS (8.5 mg, 0.039 mmol, as internal std) and MeOH (5 mL) were combined in a clear glass tube and deoxygenated by bubbling with argon for 15 minutes. The tube was then placed into a LED reactor (444 nm). The reaction was quenched after 29 h irradiation (64% conversion of 4VP by ^1^H NMR spectroscopy, *M*_n,conv_ = 37.2 kDa) by removing the glass tube from the light source and opening to air. The polymer was then diluted with methanol and twice precipitated into diethyl ether (250 mL). Lastly, the precipitate was dissolved in DI water/ethanol (50/50) and dialysed against DI water (MWCO 500–1000 Da). After freeze-drying, 1.05 g of a white powder (2) was obtained.

All polymers were analysed by GPC (DMF, LiBr 25 mM), with low to moderate dispersities (*Đ* ≤ 1.35). Successful chain extension for 2 from pDMA_261_ was supported by a shift of a single peak from 28.5 kDa to 30.0 kDa (Table S1 and Fig. S14†).

Coumarin maleimide labelled polycations were prepared using a modified one-pot aminolysis/thiol–ene procedure (full details in ESI†).^32^ Cy5 labelled 1 (1c) was prepared using an azide functionalised allyl derivative and subsequently functionalising with Cy5-alkyne *via* Cu catalysed click (full details in ESI†).

### Polyvinylpyridine (block-co)polymer ionization

To prepare the polycations used in this work 1 and 2 were reacted with either DVP (∼1 eq.) in D_2_O at room temperature for 2–3 days to yield 1a and 2a, or BuBr (∼10 eq.) in MeOH at 65 °C for ∼20 h to yield 1b and 2b. The polymers were then purified by a combination of precipitation into diethyl ether and dialysis against 100 mM NaCl and DI water. Full synthetic procedures are in ESI.†

### Polymer titration study

Throughout this work 10 mM pH 7.4 phosphate buffer is widely used and abbreviated to PB. Stock solutions of all polycations (1a, 1b, 2a, 2b) were prepared in PB at concentrations ranging from 2–20 mM (cationic units). Increasing amounts of each polycation were then added to pDNA (650 ng) in 0.97 mL PB. These additions corresponded to N/P from 0.5 to 16 (ratio of cationic nitrogens in polymer to anionic phosphates in pDNA). Total volume of polycation addition was <50 μL to keep concentration of pDNA approximately constant (0.65 μg mL^−1^). Results are presented as means ± SD (3 measurements on each sample, ran in duplicate).

### Micelle and polyplex preparation

Stock solutions of all polycations were prepared in PB to concentrations as required for the N/P of the complex (typically 6 mM polycationic units). For all experiments pDNA complexes were prepared fresh (used within day). pDNA (typically 400 μg mL^−1^, 10 mM Tris-HCl, pH 8.5 buffer), polycation and PB were combined and shaken in 1.5 mL Eppendorf tubes (final pDNA concentration = 65 ng μL^−1^). These were then briefly centrifuged to ensure full mixing of the solutions at the bottom of the tube. The complexes were incubated at room temperature for 30 min before characterisation or use. For size analysis, complexes were diluted to 6.5 ng μL^−1^ (pDNA) just before analysis, with data presented means ± SD (*n* = 3).

### Lipid and lipopolyplex preparation

The lipid DOPE/DC-Chol (70/30) was prepared according to manufacturer's instructions (Avanti). Specifically, stock solutions of DOPE and DC-Chol were prepared in chloroform, these were then combined to yield a solution of DOPE and DC-Chol at a 70/30 wt%. The chloroform was evaporated (rotary evaporation) forming a thin lipid film. The film was dissolved in DI water (sonication 5–10 minutes) at a concentration of 4 mM total lipid. The solution was passed through a 0.22 μm filter to sterilise. The size of the lipids in DI water was measured as 91 ± 9.8 nm using DLS.

The lipid solution was then added to the desired polyplex at an L/P (lipid/pDNA) mass ratio of 10 : 1. This was then diluted with PB to a final pDNA concentration of 32.5 ng μL^−1^. These were then briefly centrifuged and incubated at room temperature for 30 min before characterisation or use. For size analysis, complexes were diluted to 6.5 ng μL^−1^ (pDNA) just before analysis, with data presented means ± SD (*n* = 3). The lipid complex (Lipid) was prepared as above without the addition of a polycation and cell culture medium used as diluent rather than PB. Note: the use of cell culture medium is important, as lipid complexes prepared in PB transfected poorly (similar to naked pDNA).

### pDNA release studies

Polyplexes and micelles were prepared (65 ng pDNA per μL) then diluted into either HAMS-F12 (phenol red free), 1 mM GSH, 1 mM GSH in HAMS-F12 (phenol red free) (both) or PB to a final concentration of 6.5 ng pDNA per μL. These solutions were maintained at 37 °C for up to 24 h, with their zeta potential and size measured at specified time points. For gel electrophoresis measurements samples were taken at 1 and 4 hours and loaded into gels within 30 minutes of sampling.

### Cell culture

Chinese Hamster Ovaries cells, CHO-K1 (DSMZ) were cultured in HAMS-F12 medium (Gibco) containing 10% FBS (cell culture medium). Cells were cultured in a humidified atmosphere of 5% CO_2_ at 37 °C. Cells were passaged every 3 to 4 days up to passage 16. Detailed procedures are given in the ESI.†

### Statistical analysis

For flow cytometry data where tests of significance are given, a two-tailed unpaired Students *t*-test was applied. *P* < 0.05 was regarded as statistically significant.

## Results and discussion

### Formation of pDNA complexes

**Fig. 2:**
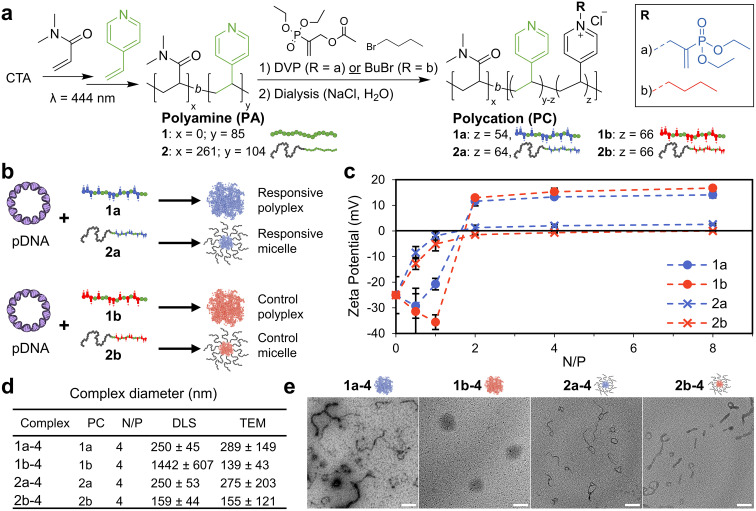
Preparation of polycations and their pDNA (pEGFP-C1) complexes. (a) Preparation of polyamines by RAFT polymerisation followed by their reaction with electrophiles to generate either responsive or permanently cationic (control) polycations. (b) Formation of micelle and polyplex type pDNA complexes studied in this work. (c) Titration of polycations to pDNA (6.5 μg mL^−1^ pDNA; 20 μM phosphates) as a function of N/P (prepared in 10 mM pH 7.4 phosphate buffer). (d) Summary of N/P = 4 pDNA complex size. Diameters are reported as the average across 3 samples (DLS) or 3 images (TEM) ± SD. For TEM, diameter is the length of the longest dimension. (e) TEM images of N/P = 4 pDNA complexes (stained, 2 wt% uranyl acetate), scale bar 200 nm. Additional characterisation data can be found in Fig. S4–14† (gel electrophoresis, polymer characterisation and additional DLS/TEM data).

To study the transfection capability of nucleophile responsive cationic PVPs, polymers with architectures capable of forming both polyplex and micelle pDNA complexes were prepared by RAFT polymerisation (Fig. 2a). For this, a 4-vinyl pyridine homopolymer (1, p4VP_85_) and block-copolymer (2, pDMA_261_-*b*-4VP_104_) were synthesised. In both cases, GPC peaks were monomodal, with molecular weights determined by ^1^H NMR conversion and GPC in reasonable agreement (1: *M*_n,NMR_ = 9.2 kDa, *M*_n,GPC_ = 5.3 kDa, *Đ* = 1.34; 2*M*_n,NMR_ = 37.2 kDa. *M*_n,GPC_ = 30.0 kDa, *Đ* = 1.30). These polymers were then ionized by reaction with diethyl(α-acetoxymethyl) vinylphosphonate (DVP), leading to the desired responsive cationic PVPs (1a and 2a, respectively, chemical structures shown in Fig. 1a). A matching set of non-responsive and permanently cationic control polymers were prepared by reaction with BuBr (1b and 2b, Fig. 1a). The conjugate acid p*K*_a_ of vinyl pyridine is 4.9,^33^ so any unreacted pyridine residues are assumed to be neutral at physiological pH (7.4). Both sets of polycations were dialysed against 0.1 M NaCl and then DI water to yield polycations with a chloride counterion. Note that without this step the responsive polycations are unstable during freeze-drying (Fig. S3†).

We then combined these polycations with pDNA (pEGFP-C1, 4.7 kbp) and studied their complex formation as a function of N/P, defined here as the ratio of cationic nitrogens (N) to anionic phosphates (P) (Fig. 2b and c). With increasing N/P, the zeta potential increased from negative values for pure pDNA (−25 ± 7 mM) to positive values, plateauing around 14 and 17 mV for 1a and 1b, indicating the formation of polyplexes with a net positive surface charge. Complexes from the block copolymers 2a and 2b recorded near neutral zeta potentials (0 and 3 mV) due to their micelle forming neutral block dominating the surface charge. Encapsulation of pDNA appears complete at N/P ≥ 2 for all polymers, a conclusion supported by retention of samples to the baseline for N/P ≥ 2 during gel electrophoresis (Fig. S4†).

Lastly, we studied the size and morphology of the pDNA complexes (N/P = 4) by both dynamic light scattering (DLS) and transmission electron microscopy (TEM, Fig. 2d and e). The responsive polyplex 1a-4 (pDNA complex designation : polymer number-N/P ratio) consisted of a mixture of spherical and short wormlike species, while the control polyplex 1b-4 was typically spherical. The pDNA micelle complexes 2a-4 and 2b-4 formed a mixture of worm-like and ring species, as is expected for cationic block-copolymer pDNA complexes.^4^ Thus, the presence of worms for 1a-4 may be due to the phosphodiester terminus of the responsive cationic moiety acting as a water interfacing group. The size of pDNA complexes was typically in the 100–250 nm range, with good agreement between DLS and TEM. In the case of 1b-4, however, the DLS diameter (1.44 μm) was found to be 10-fold larger than that observed from TEM (139 nm). We note that this disparity is most likely due to a numerically small amount of large (>1 μm) species dominating the DLS measurement (Fig. S6†).

### Intracellular and extracellular release

**Fig. 3:**
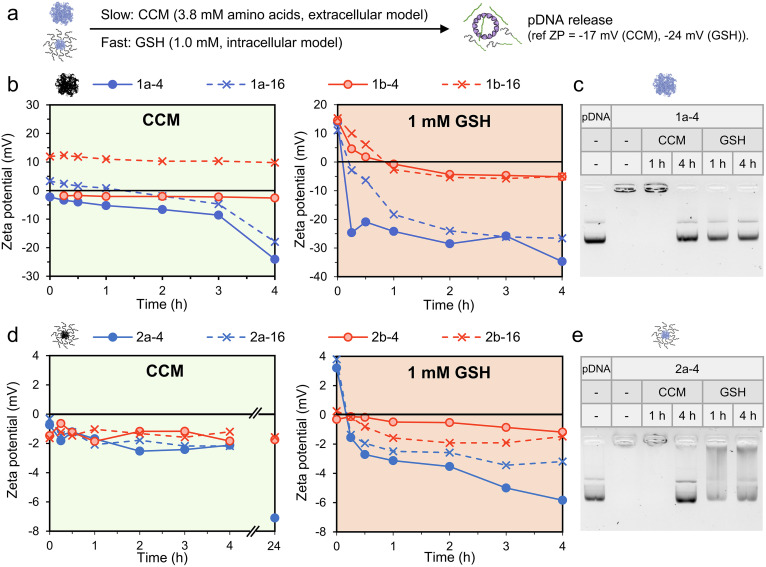
Decationization and pDNA release studies under model intra- and extra-cellular conditions. Samples were maintained at 37 °C and the concentration of pDNA (as anionic phosphates) was maintained at 0.02 mM for zeta potential (ZP) measurements and 0.06 mM for gel electrophoresis. (a) Expected responsive complex pDNA release under model extracellular (HAMS-F12 media, CCM) and intracellular (1.0 mM GSH) conditions. (b) Change in ZP over time for responsive (blue) and non-responsive (red) polyplexes in CCM and 1 mM GSH. (c) Gel electrophoresis measurements of 1a-4 after incubation in CCM or 1 mM GSH for 1 and 4 h. (d) Change in ZP over time for responsive (blue) and non-responsive (red) micelles in CCM and 1 mM GSH. (e) Gel electrophoresis measurements of 2a-4 after incubation in CCM or 1 mM GSH for 1 and 4 h. Additional gel electrophoresis data in Fig. S15 and S16.†

After forming polyplex and micelle pDNA complexes, we investigated their rate of decationization and pDNA release in conditions simulating the intra- and extra-cellular environment (Fig. 3a). Estimates for the concentration of free amino acids in human plasma range from 2.3–3.1 mM,^27,28,34^ while the concentration of GSH is typically only 2–20 μM.^3,31^ Thus, we selected serum free HAMS-F12 cell culture media (CCM) as an extracellular model, with an amino acid concentration of 3.8 mM. We note that in CCM a range of nucleophiles are present (4.7 mM RNH_2_, 0.3 mM R_2_NH and 0.2 mM RSH), all of which may be able to trigger the de-ionization reaction. The mammalian intracellular environment consists of an elevated concentration of both amino acids (∼50 mM)^29,30^ and GSH (1–10 mM).^3,31^ Given the known high reactivity of the responsive polycations towards thiols, we selected 1.0 mM GSH as a simple and conservative model. This approach also allows for a clearer understanding around the role of GSH compared to amino acids.

We studied the de-ionization process at 37 °C by both zeta potential (ZP) and gel electrophoresis across 4 h. Control complexes were found to be stable in both CCM and 1 mM GSH by gel electrophoresis, as expected (Fig. S15†). Interestingly, a reduction in zeta potential to near neutral values was typically observed for the control complexes, likely due to adsorption of ions from the media (Fig. 3, red data points).

Promisingly, the responsive complexes (Fig. 3, blue data points) yielded greater reductions in ZP for all conditions, indicating loss of cationic charge. The rate of this loss is dependent on the media, complex morphology and N/P; with low N/P polyplexes in 1 mM GSH giving the most rapid response. For example, the responsive polyplex 1a-4 recorded a gradual reduction in ZP over 4 h in CCM from −2 to −24 mV (Fig. 3b), indicating approximately complete pDNA release (ZP = −17 mV for pDNA only in CCM). In 1 mM GSH a significantly faster release was observed, with ZP reducing from +14 to −25 mV in 15 minutes (ZP = −24 mV for pDNA only in 1 mM GSH). This demonstrates a 16 times faster release of pDNA in model intracellular conditions compared to extracellular conditions for 1a-4. These conclusions were supported by gel electrophoresis, with complete release observed within 4 h and 1 h for CCM and 1 mM GSH, respectively (Fig. 3c).

Increasing N/P to 16 (1a-16) resulted in a quite similar ZP decrease in CCM, albeit starting at a more positive value (from +3.3 to −18 mV in 4 h). Interestingly, in GSH the ZP drop to −24 mV was approximately 8 times slower compared to the N/P = 4 polyplex, suggesting a significantly slower decationization and pDNA release. Note, however, that at N/P = 16 the molar excess of nucleophilic species to responsive cationic units is reduced to approximately 3 in GSH (from 12 for N/P = 4), while in CCM the excess remains greater than 10 for all N/P. Thus, the difference may at least partly be due to a reduced nucleophile excess in our model closed system.

For the micelle complexes in CCM, little change in ZP was observed over 4 h for both 2a-4 (from −0.7 to −2.1 mV) and 2a-16 (from −0.3 to −2.2 mV, Fig. 3d). To probe a possible longer release profile, an additional measurement was taken at 24 h, which recorded a significantly greater reduction for both 2a-4 (−7.1 mV) and 2a-16 (−12.5 mV). While this suggests pDNA release in the order of 24 h, gel electrophoresis measurements on 2a-4 identified release within 4 h. It is worth noting that the expected product after responsive micelle deionization (2) shifts ZP values measured for pDNA closer to zero (Table S2†). This does complicate interpretation of the ZP data, therefore the release timeframe can likely only be estimated as being between 4 and 24 h. In 1 mM GSH, the responsive micelles were similarly found to deionize slower than their polyplex counterparts. Here, however, a reduction in ZP was already clear after 4 h and gel electrophoresis measurements indicated a partial pDNA release after only 1 h (Fig. 3e).

### Cellular uptake and transfection

After demonstrating nucleophile triggered pDNA release in both 1 mM GSH and CCM, we investigated the *in vitro* transfection of EGFP encoding pDNA complexes (N/P = 4 and 16) on Chinese hamster ovary (CHO) cells. Initial studies analysed by confocal laser scanning microscopy (CLSM) indicated poor EGFP transfection for all complexes which was similar to naked pDNA (Fig. S17†). A lipid formulation (DOPE/DC-Chol : 70/30) was as used as a positive control, enabling a normalised fluorescence to be evaluated. This lipid formulation (Lipid), with a mass ratio of lipid : pDNA = 10 : 1 was analysed by DLS, yielding a size of 547 ± 111 nm. The best performing polymer complex was the non-responsive polyplex 1b-16, with a normalised fluorescence (GFP) of 0.08, however the cells were visually rounded up and shrunken. In addition, cell numbers appeared low for this formulation suggesting most of cells were detached and non-viable due to the large polycation excess (Fig. S18†).

**Fig. 4:**
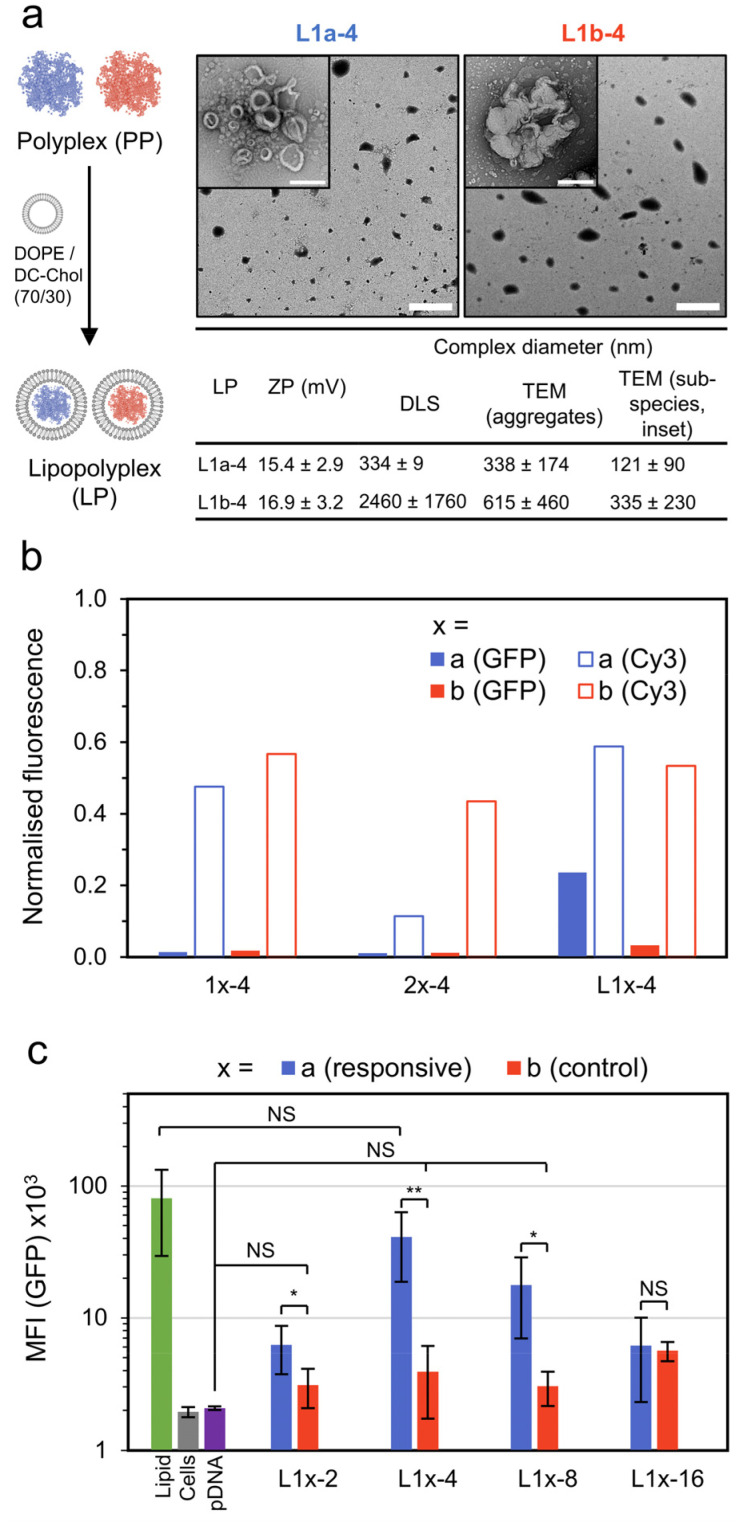
Lipopolyplex preparation, EGFP transfection and pDNA localisation data. (a) Preparation and particle characterisation of responsive and control lipopolyplexes (lipid : pDNA = 10 : 1 w/w). Lipopolyplexes are composed of aggregates (main TEM image, scale bar 2 μm) of smaller sub-species (inset image, scale bar 200 nm). (b) Normalized fluorescence intensity of CHO cells treated with various pDNA (1 μg per well) complexes as measured by CLSM. GFP fluorescence measured 48 h after treatment and Cy3 fluorescence (from Cy3 labelled pDNA, *λ*_ex_ = 543 nm, *λ*_det_ = 548–797 nm) measured 4 h after treatment. The results are normalised to a lipid formulation (DOPE/DC-Chol : 70/30). (c) Mean GFP fluorescence intensity (MFI) of CHO cells 48 h after treatment with control and responsive lipopolyplexes as measured by flow cytometry (*n* = 5, each measurement >10k cells, error bars are ±SD, NS = *p* > 0.05, * = *p* < 0.05, ** = *p* < 0.01). Additional TEM and CLSM images in Fig. S17–S21.†

To improve transfection performance, we prepared lipopolyplexes from both the responsive and control polyplexes at a mass ratio of lipid : pDNA = 10 : 1 (Fig. 4a). Again, the lipid mixture of DC-Chol/DOPE (70/30) was used, as has already been successfully applied in lipopolyplex preparations.^22^ The lipopolyplexes (N/P = 4) were characterised by DLS, yielding a size of 334 ± 9 nm for the responsive lipopolyplex (L1a-4) and 2.45 ± 1.8 μm for the control lipopolyplex (L1b-4). This difference in *Z*-ave diameter follows the same trend observed from the constituent polyplexes (1b-4 is approximately 6 times larger than 1a-4) and again may be related to the phosphodiester moiety in the responsive complexes acting as a water interfacing group, reducing aggregation. The lipopolyplexes were positively charged, with ZP values of 15.4 mV (L1a-4) and 16.9 mV (L1b-4). TEM images revealed the formation of layered or walled structures, which tended to aggregate under TEM imaging conditions (Fig. S21†). Promisingly, CHO cells treated with L1a-4 had a noticeable increase in EGFP expression with normalised GFP fluorescence more than 7 times larger than its control lipopolyplex (L1b-4, Fig. 4b).

With this promising result we investigated the cellular uptake of polycation and pDNA for all complexes to understand if cellular internalisation was limiting, particularly for the 1b-4 and L1b-4 complexes, which have a noticeably larger diameter (DLS) than the other complexes. We prepared coumarin end-functionalised derivatives of our synthesised polycations (Scheme S1†) and combined them with Cy3 labelled pDNA. The extent of pDNA and polymer internalisation in CHO cells was then evaluated 4 h after treatment using CLSM (Fig. 4b and Fig. S19†). Uptake of pDNA was found to be similar for all complexes except 2a-4, which internalised poorly. Importantly, L1a-4 and L1b-4 had normalised Cy3 fluorescence values within 10% of each other, indicating that the observed difference in transfection is not due to poor pDNA cellular entry with the control complex. A similar uptake pattern was observed for the polycations (coumarin fluorescence), as expected for complexed species (Fig. S20†). While a poor cellular uptake may have been expected for L1b-4 due to its large *Z*-ave diameter (2.45 μm), TEM image analysis shows the complex was highly disperse and includes many smaller species (≤350 nm) which may more readily internalise.

Comparing the responsive lipopolyplex (L1a-4) to its constituent polyplex (1a-4), we note only a 19% larger Cy3 fluorescence, despite its more than 16 times increase in EGFP expression. A plausible explanation may be that the polyplex (and micelle) carriers have poor endosome escape, which is overcome in lipopolyplexes where lipids such as DOPE are known to assist.^8,35^ Indeed, polymers 1 and 2 lack specific endosomal escape functionality such as amino monomers which become protonated in the acidic endosomes or lipophilic monomers.^36–40^

To better quantitate the relative performance of the responsive and control lipopolyplexes, an extensive transfection study was analysed by flow cytometry (Fig. 4c). Lipopolyplexes were prepared at N/P values ranging from 2 to 16, with the control lipopolyplexes offering no significant improvement (NS: *p* > 0.05) over cells treated with pDNA alone for all N/Ps except 16. Importantly, the responsive lipopolyplexes either significantly outperformed (N/P = 2, 4, 8) or matched (N/P = 16) their control counterparts. At N/P = 4 the greatest improvement was observed, with a GFP mean fluorescence intensity (MFI) 10 times greater than its control and a MFI that was insignificantly different from the lipid.

### Role of nucleophile responsive polycation

Such an improvement in transfection performance for the responsive lipopolyplex compared to a permanently cationic control is promising, however it did not outperform its constituent lipid on its own. This clearly limits applications of the current responsive lipopolyplex, however we believe future application of the charge-reversing chemistry in more optimised designs may overcome this. For example, incorporation of lipophilic monomers such as butyl acrylate has been shown to improve transfection in charge-reversing polymer systems.^39,40^ For the purposes of this study, we wish to demonstrate that the improved transfection is not simply due to extracellular deionization of the lipopolyplex into the constituent lipid complex. Specifically, we wanted to show that the responsive polycationic unit reaches the intracellular milieu intact and is then able to undergo deionization to release the bound pDNA.

**Fig. 5:**
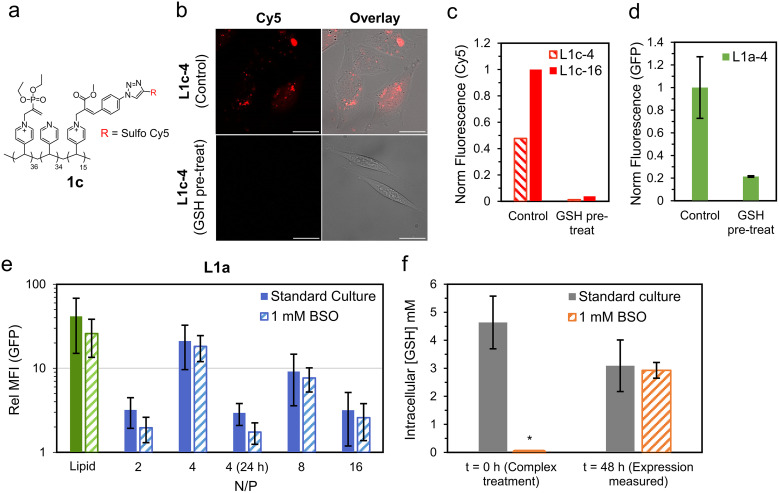
Investigations into role of nucleophile responsive polycationic functionality. (a) Polymer structure of 1c, a Cy5 labelled responsive polycation. (b) Cy5 (red) localisation study of CHO cells treated with lipopolyplexes prepared using 1c (L1c-4, Control). These are compared to cells treated with matching lipopolyplexes prepared from 1c pre-treated with 5× excess GSH (L1c-4, GSH pre-treat). Images were taken using CLSM with Cy5 (*λ*_ex_ = 633 nm, *λ*_det_ = 638–797 nm, red) and bright field channels overlayed, scale bar is 25 μm. (c) Normalised Cy5 fluorescence of cells treated with lipopolyplexes prepared from 1c (Control) and decationized 1c (GSH pre-treat). (d) Normalised EGFP transfection of cells treated with L1a-4 (Control) compared to L1a-4 with GSH pre-treatment (2 mM GSH in CCM, 37 °C for 1 h). Data is mean ± SD (*n* = 3) as evaluated by flow cytometry. (e) GFP fluorescence 48 h (or 24 h where stated) of lipopolyplex treated CHO cells relative to untreated cells under the same culture condition (standard culture or 1 mM BSO). Data is mean ± SD (*n* ≥ 3) as evaluated by flow cytometry. (f) Intracellular GSH concentration of standard culture and BSO treated cells at *t* = 0 and 48 h. Data is mean ± SD (*n* = 4) as measured using a commercial luminescence-based assay which produces luciferase in the presence of GSH (Fig. S28†). *For *t* = 0 (1 mM BSO) intracellular GSH was below limit of detection.

We began by synthesising a new variant of polymer 1a (1c, Fig. 5a), with a fraction of the responsive cationic moieties replaced with a Cy5 labelled analogue (see ESI† for full synthetic details). NMR studies on 1c indicated similar decationization behaviour to 1a when exposed to an excess of a model amino acid (threonine, Fig. S30†). Promisingly, cells treated with lipopolyplexes prepared from 1c (N/P = 4 and 16) had noticeable intracellular Cy5 fluorescence 4 h after treatment (Fig. 5b and c). To account for any internalisation of the free Cy5 species formed after deionization, a decationized sample of 1c was prepared by treating it with 5× excess GSH. Cells treated with the decationized 1c (GSH pre-treat) recorded little Cy5 fluorescence (<4% of the corresponding L1c complex), indicating that the free Cy5 moiety internalises poorly. These experiments strongly indicate that the responsive cationic moiety does reach the intracellular milieu intact.

To investigate this further, we ran additional EGFP transfection studies comparing the responsive lipopolyplex L1a-4 to L1a-4 which has been pre-treated with 2 mM GSH in CCM for 1 h at 37 °C (GSH pre-treat). This treatment is intended to induce significant ‘extracellular’ deionization. In this way, if the responsive lipopolyplex derives its transfection capability from extracellular degradation, we would expect similar or enhanced transfection performance. Promisingly, we observed a greater than 75% reduction in transfection for the deionized L1a-4 (GSH pre-treat) compared to non-treated L1a-4 (control, Fig. 5d). Size analysis after this treatment demonstrated a near 50% reduction in diameter for L1a-4 and a drop in zeta potential to negative values (−32.6 mV), which was not observed for L1b-4 under the same treatment (Fig. S27†).

After demonstrating internalisation of responsive polycationic functionality, we next studied the role of intracellular GSH. For this, we repeated the EGFP expression study on cells treated with buthionine sulfoximine (BSO), which is known to inhibit GSH production.^41^ Surprisingly, BSO treated cells had little change in EGFP expression relative to untreated cells, with only the N/P = 2 lipopolyplex recording a near significant reduction (*p* = 0.11) in expression (Fig. 5e). To confirm intracellular GSH levels had reduced after BSO treatment, a luciferin based GSH assay was applied. We measured GSH levels immediately after BSO treatment, corresponding to the time when pDNA complexes are added to the cells (*t* = 0), and after a further 48 h, when the cells would be harvested for transfection analysis (*t* = 48 h). At *t* = 0, intracellular GSH for standard culture conditions was found to be within the expected range (4.6 mM), while cells treated with 1 mM BSO had significantly reduced intracellular GSH (below limit of test detection, Fig. 5f). After 48 h, intracellular GSH was no longer depleted in BSO treated cells, with standard culture (3.1 ± 0.9 mM) and BSO (2.9 ± 0.3 mM) recording similar GSH concentrations. This recovery in intracellular GSH clearly limits the conclusions that can be drawn from the 48 h transfection measurements, and thus EGFP expression after only 24 h was studied for the N/P = 4 complex (Fig. 5e). At 24 h, EGFP expression was significantly reduced compared to 48 h, and while relative GFP fluorescence in standard culture (2.9 ± 0.9) was larger compared to BSO treated cells (1.7 ± 0.9), this difference was again found to be insignificant (*p* = 0.21).

The mismatch of timescales around GSH depletion and EGFP transfection does not allow for definitive conclusions from this experiment. What is most likely, however, is that given the high concentration of intracellular GSH and amino acids (∼50 mM), a combination of both trigger polymer decationization and pDNA release. Such a conclusion is also supported by our model pDNA release studies, which demonstrate release within 4 h for both 1.0 mM GSH and 3.8 mM amino acids.

### Cytotoxicity of polycations and lipopolyplexes

**Fig. 6:**
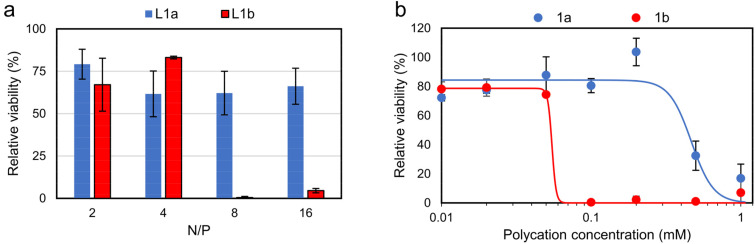
*In vitro* cytotoxicity studies on CHO cells *via* MTS assay (mean ± SD, *n* = 3). Cells were treated with (a) lipopolyplexes (0.5 μg pDNA per well) at various N/P ratios and (b) responsive and non-responsive polycations. Data is fitted using sigmoidal four-parameter logistic model, with EC_50_ determined as 0.47 mM for 1a and 0.05 mM for 1b, see Fig. S32† for full description of fitting parameters. Note: at N/P = 4 lipopolyplexes contain a polycation concentration of 0.06 mM.

The *in vitro* cytotoxicity of the lipopolyplexes and constituent polycations was evaluated *via* an MTS assay (Fig. 6). Promisingly, viability for cells treated with responsive lipopolyplexes remained ≥60%, regardless of N/P, while non-responsive lipopolyplexes were notably toxic for N/P ≥ 8 (viability < 5%). Cells treated with naked polycations demonstrated a similar trend, with 1b reducing viability to 0.4% at 0.1 mM (23 μg mL^−1^). This concentration of 1b correlates with the concentration of 1b in wells treated with its N/P = 8 lipopolyplex (0.12 mM), indicating a matching onset of toxicity for both the naked polymer and lipopolyplex. By fitting the data with a sigmoidal model, a near 10 times larger effective concentration of half maximal effect (EC_50_) is obtained for 1a (0.47 mM) compared to 1b (0.05 mM). Given that 1a can be a product in a DVP fuelled chemical reaction network (CRN), we found its good biocompatibility noteworthy and in large contrast to commonly used reagents in typical CRNs.^42–45^ We also analysed the toxicity of DVP and found it non-toxic (82% viability) at 0.24 mg mL^−1^ (1 mM) (Fig. S31†).

## Conclusions

We studied how biological nucleophiles can be used to trigger intracellular DNA release from charge-reversing polycations, leading to improved transfection and reduced cytotoxicity. The cationic-to-neutral transition is rapidly triggered with glutathione (a thiol), while amino acids (primary amines) slowly deionize the polymer. Micelle, polyplex and lipopolyplex type pDNA complexes were prepared, with lipopolyplexes found to effectively transfect CHO cells. These lipopolyplexes yielded up to 10 times more transfection than analogous non-responsive lipopolyplexes and showed reduced cytotoxicity. This improved transfection was achieved despite a similar cellular internalisation of pDNA for both treatments, indicating the importance of polymer decationization and pDNA release. To understand this behaviour, we studied the charge-reversing process in model extracellular (amino acid rich cell culture media) and intracellular (1 mM glutathione) conditions. Polyplexes were found to deionize and release their pDNA cargo 16 times faster (within 15 minutes) in model intracellular conditions compared to extracellular conditions. We thus hypothesise that the nucleophile triggered decationization, which is significantly accelerated in the intracellular space, leads to pDNA release for improved transfection and reduced cytotoxicity.

## Author contributions

R. L.: conceptualisation, investigation, writing – original draft. A. M.: investigation, writing – review & editing. B. K.: investigation (DVP and Me-Az synthesis). P. B.: resources, supervision, writing – review & editing. R. E: conceptualisation, resources, supervision, writing – review & editing.

## Conflicts of interest

There are no conflicts of interest.

## Supplementary Material

PY-014-D3PY00075C-s001
