# Supplementary material for: Nucleophile responsive charge-reversing polycations for pDNA transfection
Source: Polym Chem. 2023 Mar 9;14(14):1591–601. doi: 10.1039/d3py00075c (PMC10071491; doi:10.1039/d3py00075c)
Supplement: PY-014-D3PY00075C-s001 [file PY-014-D3PY00075C-s001.pdf]

Electronic Supplementary Material (ESI) for Polymer Chemistry.
This journal is © The Royal Society of Chemistry 2023

Supporting information

Nucleophile responsive charge-reversing polycations for pDNA transfection

Reece W. Lewis[a*], Aswin Muralidharan[abc], Benjamin Klemm[a], Pouyan E. Boukany[a], Rienk Eelkema[a*]

[a]Department of Chemical Engineering, Delft University of Technology, 2629 HZ Delft, The Netherlands.

[b]Department of Bionanoscience, Delft University of Technology, 2629 HZ Delft, The Netherlands.

[c]Kavli Institute of Nanoscience, Delft University of Technology, 2629 HZ Delft, The Netherlands.

* Correspondence to: R.W.Lewis@tudelft.nl; R.Eelkema@tudelft.nl

Additional experimental details

Table S1. Polyvinylpyridine (block-co)polymer synthesis and characterisation data.

Polymer	CTA	$[CTA]_0$: $[4VP]_0$: $[DMA]_0$	Polym time (h)	NMR Conv (%)	Structure	$M_{n,NMR}$ (kDa)	$M_{n,GPC}$ (kDa)	$Đ$
**1**	DDMAT	1:120:0	11	71	p(4VP$_{85}$)	9.2	5.3	1.34
**pDMA$_{261}$**	CETCPA	1:0:300	2.0	87	p(DMA$_{261}$)	26.2	28.5	1.14
**2**	pDMA$_{261}$	1:160:0	29	64	p(4VP$_{104}$-*b*-DMA$_{261}$)	37.2	30.0	1.30

Radical polymerisation conversion (ρ) was calculated by monitoring reduction in the ^{1}H NMR integrals of the monomer unsaturated protons (∫M: 5.6 – 6.7 ppm for DMA, 5.5 – 6.7 ppm for 4VP) and aromatic protons in case of 4VP (7.5 ppm) relative to the internal standard DSS (0 ppm, **Equation S1**).

$$\rho = \frac{\int M(t0) - \int M(t)}{\int M(t0)} \qquad \textbf{Eq. S1}$$

For a polymerisation containing z monomers, $M_{n,NMR}$ was calculated according to **Equation S2**. Here $[M_x]_0$ is the initial concentration of monomer x, $[CTA]_0$ is the initial chain transfer agent (CTA) concentration and M_{Mx} and M_{CTA} are the monomer x and CTA molecular weights, respectively.

$$M_{n,NMR} = \sum_{x=1}^{z} \rho_x \times \frac{[M_x]_0}{[CTA]_0} \times M_{Mx} + M_{CTA} \qquad \textbf{Eq. S2}$$

Polyvinylpyridine (block-co)polymer ionization

1a. The responsive polycation **1a** was prepared by combining **1** (8.5 mg, 77 µmol 4VP) and DVP (21 mg, 89 µmol) in 0.75 mL D_2O. The suspension was shaken overnight, transforming into an intensely green coloured solution, indicating formation of the polycationic species. After 48 h the solution was dialysed against 100 mM NaCl (30 mL, 3x) and DI water (30 mL, 3x). A green solid (12.3 mg) was then obtained after freeze-drying the retentate, with degree of cationization determined as 63% by ^1H NMR (D_2O) (**Figure S9**), $M_{n,NMR}$ = 20.7 kDa.

2a. The responsive polycation **2a** was prepared by combining **2** (38 mg, 106 mmol 4VP) and DVP (25 mg, 106 mmol) in 1.0 mL D_2O. The solution was heated (not required but accelerates reaction) to 45°C. After 65 h dialysed the solution against 100 mM NaCl (30 mL, 3x) and DI water (30 mL, 3x). A green solid (40 mg) was then obtained after freeze-drying the retentate, with degree of cationization determined as 61% by ^1H NMR (D_2O) (**Figure S10**), $M_{n,NMR}$ = 50.6 kDa.

1b. The non-responsive control polycation **1b** was prepared by combining **1** (200 mg, 1.84 mmol 4VP) and BuBr (2.0 mL, 18 mmol) in 2.0 mL anhydrous MeOH. The solution was heated to 65°C and stirred for 17 h, followed by precipitation into diethyl ether (200 mL). The solid was collected which was dissolved in 100 mM NaCl and dialysed against 100 mM NaCl (100 mL, 3x) and DI water (100 mL, 3x). Freeze-dried to 76 mg red solid, with degree of cationization determined as 77% by ^1H NMR (D_2O) (**Figure S12**), $M_{n,NMR}$ = 15.4 kDa.

2b. The non-responsive control polycation **2b** was prepared by combining **2** (400 mg, 1.1 mmol 4VP) and BuBr (1.2 mL, 11 mmol) in 5.0 mL anhydrous MeOH. The solution was heated to 65°C and stirred for 22 h followed by precipitation into diethyl ether (200 mL). The white solid was collected and dissolved in DI water, then dialysed against 100 mM NaCl (100 mL, 3x) and DI water (100 mL, 3x). Freeze-dried to 325 mg red/brown solid, with degree of cationization determined as 63 % by ^1H NMR (D_2O) (**Figure S13**), $M_{n,NMR}$ = 43.2 kDa.

Note: both responsive and non-responsive type polycations are stable on a timescale of weeks in refrigerated aqueous solutions at neutral pH (**2a** showed no change in cationic substitution after 2 weeks when stored in the fridge). However, the freeze-dried solid of **1a** and **2a** is apparently unstable and can crosslink if stored for ~1 week or more (does not redissolve, only swells).

Additionaly, the polycationic derivatives of 1 and 2 were found to have strong interactions with the GPC column, thus $M_{n,GPC}$ for the polycations was unable to be measured.

Fluorescently labelled polycations

1 and **2** were labelled with coumarin as a fluorescent tracer to allow for investigation into polymer cellular internalisation of their cationic derivatives. These were prepared using a one-pot aminolysis/thiol-ene procedure according to **Scheme S1**.

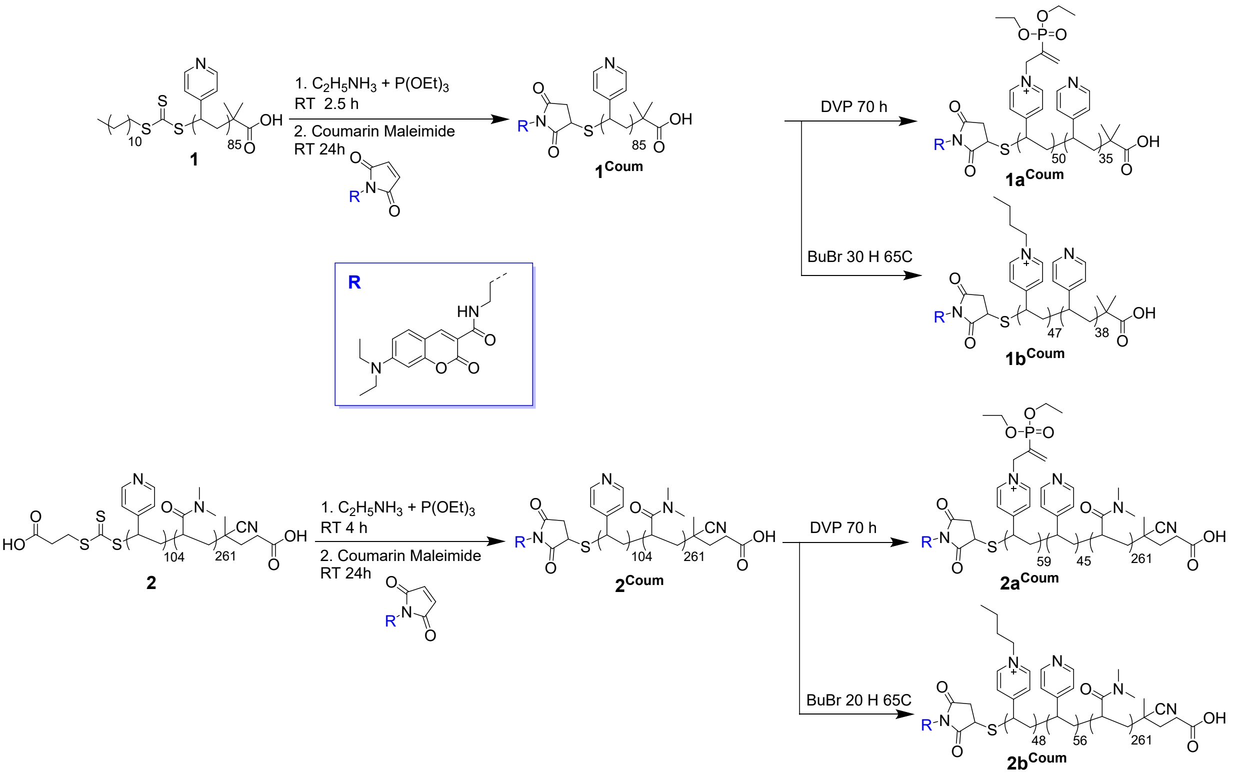

Scheme S1. Preparation of coumarin labelled polycations. Scheme is simplified for clarity, with non-functionalised and side product species not shown.

1^{coum}. **1** (130 mg, 11 μmol pyridine units) was dissolved in 1.5 mL THF and 0.25 mL DMF. The yellow solution was then deoxygenated by argon bubbling for 5 minutes. Ethylamine (120 μL of 2 M solution in THF, 212 μmol) and $P(OEt_3)$ (20 μL, 120 μmol) were added, resulting in a solution colour change from yellow to brown. After stirring for 2.5 h at RT, Coumarin maleimide (11.2 mg, 29 μmol) was added and the solution was left stirring overnight. To purify, precipitated into

diethyl ether (200 mL) and collected a yellow solid. This solid was twice washed with DI water, then redissolved in ethanol and precipitated again into diethyl ether. The solid was dried to 115 mg, with ^{1}H NMR confirming absence of remaining small molecule species and UV-Vis spectroscopy (ε = 49,000 cm^{-1} M^{-1} in MeOH) determined extent of end-group functionalisation to be approximately 81%.

1a$^{\mathbf{coum}}$. This was prepared using analogous method described for preparation of **1a** by replacing **1** with **1**$^{\mathbf{coum}}$. Degree of ionization was determined to be 58% by ^{1}H NMR (**Figure S22**), $M_{n,NMR}$ = 19.9 kDa.

1b$^{\mathbf{coum}}$. This was prepared using analogous method described for preparation of **1b** by replacing **1** with **1**$^{\mathbf{coum}}$. Degree of ionization was determined to be 55% by ^{1}H NMR (**Figure S23**), $M_{n,NMR}$ = 13.8 kDa.

2$^{\mathbf{coum}}$. **2** (100 mg, 2.7 μmol pyridine units) was dissolved in 1.0 mL THF and 0.75 mL dioxane. The hazy yellow solution was then deoxygenated by argon bubbling for 5 minutes. Ethylamine (25 μL of 2 M solution in THF, 50 μmol) and $P(OEt_3)$ (10 μL, 60 μmol) were then added, resulting in slight loss of yellow colour in solution. After stirring for 2.5 h at RT, Coumarin maleimide (4.5 mg, 12 μmol) was added and the solution was left stirring overnight. To purify, precipitated into diethyl ether (200 mL) and collected a yellow solid. This solid was dissolved in DI water and dialysed against DI water 3 times. Freeze-dried the retentate to 77 mg, with ^{1}H NMR confirming absence of remaining small molecule species. UV-Vis spectroscopy (ε = 49,000 cm^{-1} M^{-1} in MeOH) determined extent of end-group functionalisation to be approximately 53%.

2a$^{\mathbf{coum}}$. This was prepared using analogous method described for preparation of **2a** by replacing **2** with **2**$^{\mathbf{coum}}$. Degree of ionization was determined to be 57% by ^{1}H NMR (**Figure S24**), $M_{n,NMR}$ = 49.9 kDa.

2b$^{\mathbf{coum}}$. This was prepared using analogous method described for preparation of **2b** by replacing **2** with **2**$^{\mathbf{coum}}$. Degree of ionization was determined to be 49% by ^{1}H NMR (**Figure S25**) $M_{n,NMR}$ = 42.0 kDa.

The responsive polycation **1c** with fluorescently (Cy5) labelled cationic moieties was prepared according to **Scheme S2**. The azide functionalised allyl methyl ester (**ME-Az**) used in the preparation of **1c** was synthesised according to **Scheme S3**.

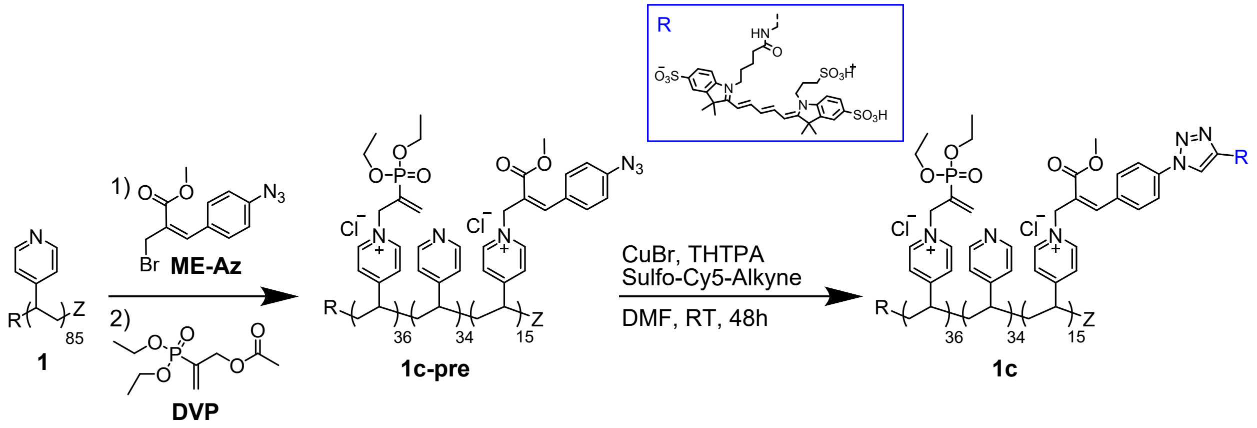

Scheme S2. Synthesis of fluorescently (Cy5) labelled polycation **1c.** Note: first reaction includes a dialysis step exchanging counterions to Cl^- as shown.

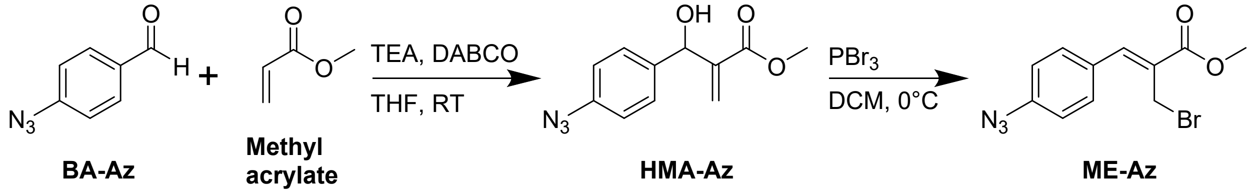

Scheme S3. Synthesis of azide functionalised allyl methyl ester (**ME-Az**) used in preparation of **1c**.

BA-Az (4-Azidobenzaldehyde). A mixture of 4-formylphenylboronic acid (33.0 mmol, 4.95 g, 1.0 eq.), sodium azide (89.2 mmol, 5.8 g, 2.7 eq.) and copper(II)acetate (3.3 mmol, 0.6 g, 0.1 eq.) are stirred for 24 hours in methanol (180 mL). After completion the reaction mixture is concentrated on celite under reduced pressure and purified by silica column chromatography (5:5 petroleum ether:ethyl acetate) to yield the title compound as a yellow oil (25.1 mmol, 3.7 g, 76%). **^{1}H NMR** (400 MHz, $CDCl_3$) δ 9.86 (s, 1H), 7.80 (d, J = 8.5 Hz, 2H), 7.08 (d, J = 8.4 Hz, 2H). **^{13}C NMR (**101 MHz, $CDCl_3$) δ: 190.6, 146.4, 133.3, 131.6, 131.6, 119.6.

HMA-Az (methyl 2-((4-azidophenyl)(hydroxy)methyl)acrylate). 4-Azidobenzaldehyde (25.1 mmol, 3.7 g, 1.0 eq.), methyl acrylate (75.4 mmol, 6.8 mL, 3.0 eq.), triethanolamine (20.1 mmol, 3.0 g, 0.8 eq.) and DABCO (25.1 mmol, 2.8 g, 1.0 eq.) are added to a flask with 20 mL THF and stirred at RT for 4 days. After completion the reaction mixture is diluted with water and extracted three times with DCM. The organic layers are then dried with Na_2SO_4 and concentrated under reduced pressure. The residue is then purified by silica column chromatography (8:2 petroleum

ether:ethyl acetate) to yield the title compound as pale yellow oil (8.1 mmol, 1.9 g, 32%). **^{1}H NMR** (400 MHz, $CDCl_3$) δ: 7.26 (d, J = 8.5 Hz, 2H), 6.90 (d, J = 8.2 Hz, 2H), 6.24 (s, 1H), 5.77 (s, 1H), 5.43 (s, 1H), 3.62 (s, 3H), 3.25 (s, 1H). **^{13}C NMR (**101 MHz, $CDCl_3$) δ: 166.6, 141.8, 139.5, 138.1, 128.1, 126.0, 119.0, 72.5, 52.0.

ME-Az ((Z) methyl 2-((4-azidophenyl)(hydroxy)methyl)acrylate). Methyl 2-((4-azidophenyl)(hydroxy)methyl)acrylate (8.1 mmol, 1.9 g. 1.0 eq.) is dissolved in anhydrous DCM (40 mL) and cooled to 0 degrees. PBr_3 (1.0 M DCM solution, 1.33 mL, 0.9 eq.) is then added dropwise under an argon atmosphere. After completion the reaction is stopped by adding ice. The mixture is then extracted with DCM and washed twice with water. The organic layers are then dried with Na_2SO_4, filtered and concentrated under reduced pressure. The residue is then purified by silica column chromatography (9.5:0.5 petroleum ether:ethyl acetate) to yield methyl 3-(4-azidophenyl)-2-(bromomethyl)acrylate as white-yellowish solid (1.9 g, 6.4 mmol, 80%). **^{1}H NMR** (400 MHz, $CDCl_3$) δ: 7.70 (s, 1H), 7.57 – 7.49 (m, 2H), 7.09 – 7.01 (m, 2H), 4.32 (s, 2H), 3.81 (s, 3H). **^{13}C NMR** (101 MHz, $CDCl_3$) δ: 166.6, 141.9, 141.6, 131.5, 130.9, 128.2, 119.5, 52.5, 26.7.

1c-pre. 1 (4.4 mg, 40 μmol 4VP), **ME-Az** (2.0 mg, 6.8 μmol), d_8-THF (0.65 mL) and D_2O (0.15 mL) were combined and stirred for 4 h. At this time ^{1}H NMR confirmed reaction of **ME-Az** with **1** (~ 18% of VP units). DVP (9.9 mg, 42 μmol) was then added and left to react for a further 110 h. The product was dialysed against 100 mM NaCl (30 mL, 3x) and DI water (30 mL, 3x). Freeze-dried to 3.9 mg, with degree of cationization determined at to be 18% (ME-Az) and 42% (DVP) by ^{1}H NMR (D_2O) (**Figure S28**).

1c. Stock solutions of CuBr (8 mg/mL) and THTPA (24 mg/mL) were prepared in DMF. THTPA (0.4 mL stock) and CuBr (0.4 mL stock) were combined and bubbled with argon for 5 minutes. The solution was briefly sonicated (to dissolve completely CuBr). 240 μL of this solution (7.2 μmol CuBr, 7.2 μmol THTPA), **1c-pre** (2 mg, 1.4 μmol azide functionality), Sulfo-Cy5-Alkyne (1.0 mg, 1.0 μmol) and 0.3 mL DMF were combined and further deoxygenated by argon bubbling for 5 minutes. The reaction was then sealed and left stirring for 72 h. To purify, the solution was dialysed against DI water (30 mL, 2x), 50 mM EDTA (30 mL, 2x) and DI water (30 mL, 3x). During initial dialysis some blue coloration from the Cy5 dye was observed in the permeate, however an intense blue coloration remained in the retentate after complete dialysis, indicating successful Cy5 functionalisation.

Cell culture (transfection)

For GFP transfection (confocal), cells were seeded in 8-well glass bottomed plates at a density of 1.5 x 10^4 cells per well, with 0.2 mL cell culture medium and incubated overnight. The medium was then replaced by fresh cell culture medium (0.2 mL) and pDNA complex solutions were added (15 µL for polyplexes and micelles, and 30 µL for lipopolyplexes). Each treatment delivered 1.0 µg pDNA / well (5 µg/mL). The cells were then incubated for 4 h, after which the medium was replaced with 0.2 mL fresh cell culture medium and incubated for a further 44 h. The cells were then imaged on a confocal laser scanning microscope (CLSM, Zeiss LSM 710) with a x20 objective (Plan-Apochromat 20x/0.8 M27, Zeiss) at λ_{ex} = 458 nm and detection range (λ_{det} = 493 – 797 nm) for GFP. Each well was imaged 4 times in both bright field and GFP fluorescent channels. The images were processed using Fiji (Image J) software, with cells sectioned manually in bright field and the area average fluorescence across all cells evaluated (≥22 cells imaged per treatment). Values are reported as normalised fluorescence which was evaluated according to **Equation S3**. Here FL_x is fluorescence for treatment x, FL_{cells} is fluorescence for untreated cells and FL_{max} is largest average fluorescence recorded.

$$Normalised\ fluourescence = \frac{FL_x - FL_{Cells}}{FL_{max} - FL_{Cells}} \qquad \textbf{Eq. S3}$$

For GFP transfection (flow cytometry), cells were seeded in 24-well plates at a density of 4.0 x 10^4 cells per well, with 0.8 mL cell culture medium. For BSO experiments the cell culture medium included 1.0 mM BSO. After incubating overnight, the medium was replaced by fresh cell culture medium (no BSO, 0.8 mL) and pDNA complex solutions were added (for polyplexes and micelles 15 µL and lipopolyplexes 30 µL). Each treatment delivered 1.0 µg pDNA / well (1.25 µg/mL). The cells were then incubated for 4 h, after which the medium was replaced with 0.8 mL fresh cell culture medium and incubated for a further 44 h. The medium was then removed and the cells were detached with Trypsin-EDTA. The cells were collected and re-suspended in 400 µL flow cytometry buffer (PBS with 2 mM EDTA, 0.5% BSA), with GFP expression quantitatively measured by flow cytometry (BD FACSCelesta Cell Analyzer, >10 000 cells counted per measurement, 5 measurements per treatment). Results were analysed and gated to single cells using FlowJo software, data is presented as mean fluorescence intensity (MFI). For comparisons between standard culture and BSO treatment, data is presented as relative MFI (Rel MFI, mean

fluorescent signals of treated cells divided by untreated cells for standard culture or BSO treatment). This was done as BSO treatment increased GFP autofluorescence of cells.

For GSH analysis, cells were seeded in 24-well plates at a density of 4.0 x 10^4 cells per well, with 0.8 mL cell culture medium. For BSO experiments the cell culture medium included 1.0 mM BSO. After incubating overnight, the medium was removed and 't = 0' cells were detached with Trypsin-EDTA and re-suspended into PBS for GSH analysis, while 't = 48 h' cells had fresh cell culture medium added (no BSO, 0.8 mL). The 't = 48 h' cells were then incubated for 4 h, after which the medium was replaced with 0.8 mL fresh cell culture medium and incubated for a further 44 h. The medium was then removed, and the cells were detached with Trypsin-EDTA and re-suspended into PBS for GSH analysis. GSH concentration was determined using a luminescence assay (GSH-Glo™ Glutathione Assay, Promega) according to manufacturer's instructions for mammalian cells in suspension. Glutathione standard curve was constructed using serial dilutions of known concentration of GSH from 5.0 – 0.04 µM (0.5 – 0.004 nmol / well) in duplicate at both time points (see *Figure S27*). Cell suspension measurements (5.0 x 10^3 cells, n = 4) of GSH were converted to approximate intracellular GSH concentration by assuming a cell volume of 1.7 pL.[38]

Cell culture (cellular uptake)

pEGFP pDNA was labelled with Cy3 (LabelIT Mirus Bio, $pDNA^{Cy3}$) according to manufacturer's instructions. Cells were seeded in 8-well glass bottomed plates at a density of 1.5 x 10^4 cells per well, with 0.2 mL cell culture medium and incubated overnight. The medium was then replaced by fresh cell culture medium (0.2 mL) and pDNA complex solutions were added (15 µL for polyplexes and micelles, and 30 µL for lipopolyplexes; all prepared using $pDNA^{Cy3}$ and coumarin labelled polymers: $\mathbf{1a^{coum}}$, $\mathbf{1b^{coum}}$, $\mathbf{2a^{coum}}$, $\mathbf{2b^{coum}}$). Each treatment delivered 1.0 µg pDNA / well (5 µg/mL). The cells were then incubated for 4 h, after which the cells were washed three times with cell culture medium. The cells were then imaged with CLSM (Zeiss LSM 710) using a x40 objective (Fluar 40x/1.30 Oil M27, Zeiss) with λ_{ex} = 405 nm, λ_{det} = 410 – 538 nm for coumarin and λ_{ex} = 543 nm, λ_{det} = 548 – 797 nm for Cy3. Each well was imaged at least 3 times in both bright field and Coumarin/Cy3 fluorescent channels. The images were processed using Fiji (Image J) software, with cells sectioned manually in bright field and the area average fluorescence across all cells evaluated in Coumarin and Cy3 channels.

For uptake of responsive cationic moiety study, cells were seeded in 8-well glass bottomed plates at a density of 1.5 x 10^4 cells per well, with 0.2 mL cell culture medium and incubated overnight. The medium was then replaced by fresh cell culture medium (0.2 mL) and pDNA complex solutions were added (30 µL, lipopolyplexes prepared using pEGFP and the Cy5 labelled polymer **1c**. Each treatment delivered 1.0 µg pDNA / well (5 µg/mL). The cells were then incubated for 4 h, after which the cells were washed three times with cell culture medium. The cells were then imaged with CLSM (Zeiss LSM 710) with a x40 objective (Fluar 40x/1.30 Oil M27, Zeiss) at λ_{ex} = 633 nm, λ_{det} = 638 – 797 nm for Cy5. Each well was imaged at least 3 times in both bright field and Cy5 fluorescent channels. The images were processed using Fiji (Image J) software, with cells sectioned manually in bright field and the area average fluorescence across all cells evaluated in Cy5 channels. Values are reported as normalised fluorescence which was evaluated according to **Equation S3**.

Cell culture (cytotoxicity)

Cytotoxicity was evaluated using a commercial MTS assay according to manufacturer's instructions (CellTiter 96® AQ_{ueous} One Solution Cell Proliferation Assay, Promega). Cells were seeded in 96-well plates at a density of 5.0 x 10^3 cells per well, with 0.1 mL cell culture medium and cultured overnight. The medium was then replaced by fresh cell culture medium (0.1 mL) and pDNA complex solutions were added (for polyplexes 7.7 µL and lipopolyplexes 15.4 µL). Each treatment delivered 0.5 µg pDNA / well (5 µg/mL). The cells were then incubated for 48 h, after which 20 µL CellTiter 96® AQ_{ueous} One Solution Reagent was added to each well, with the cells incubated for a further 2 h. The absorbance at 490 nm was then measured for each well using a plate reader and each treatment was measured in triplicate. Cell viability was then evaluated by subtracting the absorbance recorded for no cells and dividing this value by that from untreated cells.

Additional figures

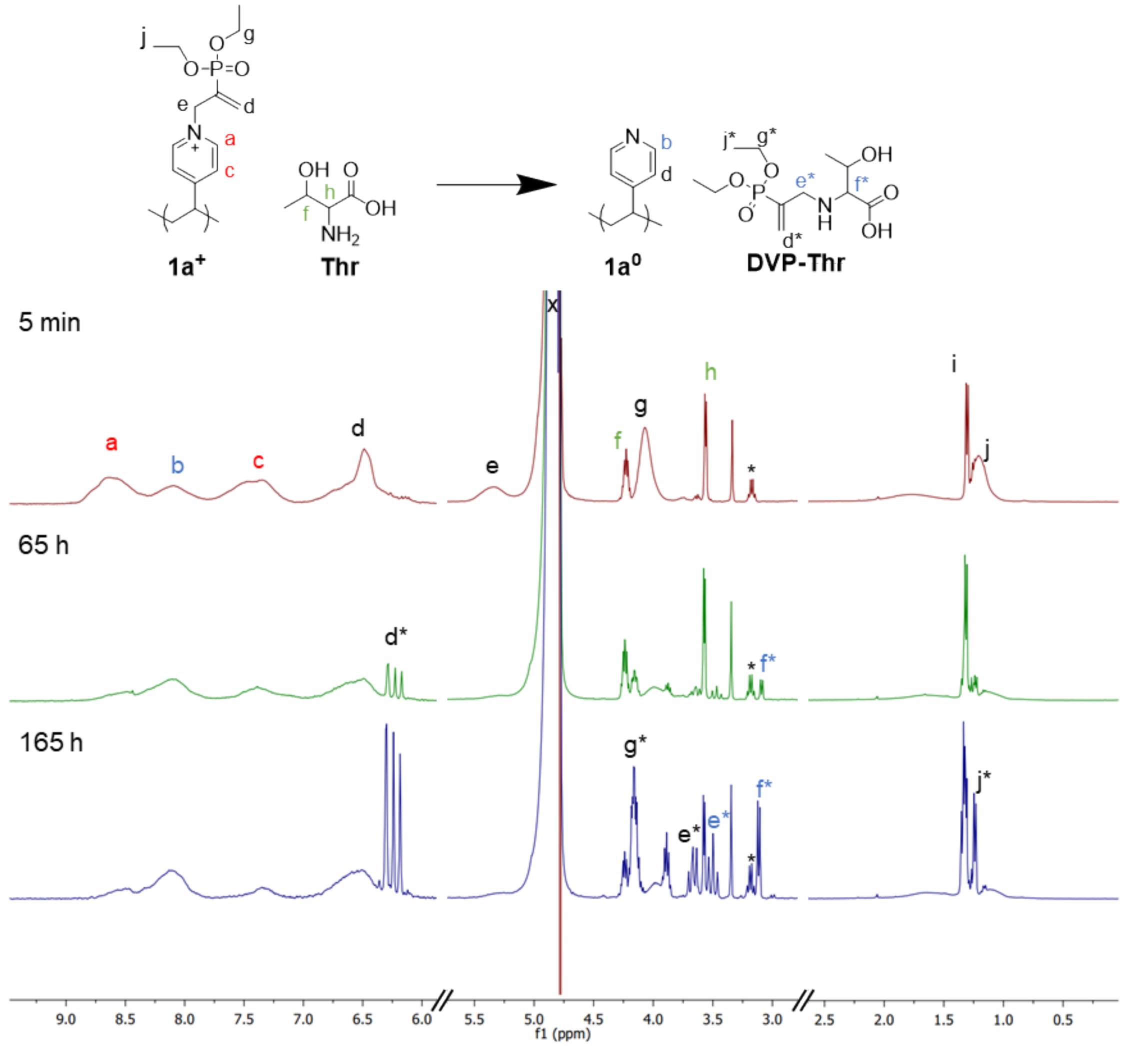

Figure S1. Example ^{1}H NMR (D_2O) spectra from reaction of cationic units on **1a** (**1a**$^{+}$) with threonine (**Thr**) to form the neutralised units on **1a** (**1a**^{0}) and side product **DVP-Thr**. Spectra split into 3 differently scaled sections for clarity. Integrals were standardised to an ethanol internal standard (*), with cationic conversion evaluated based on red (**1a**$^{+}$), and blue (**1a**^{0}) signals. See **Figure 1** for kinetic trace.

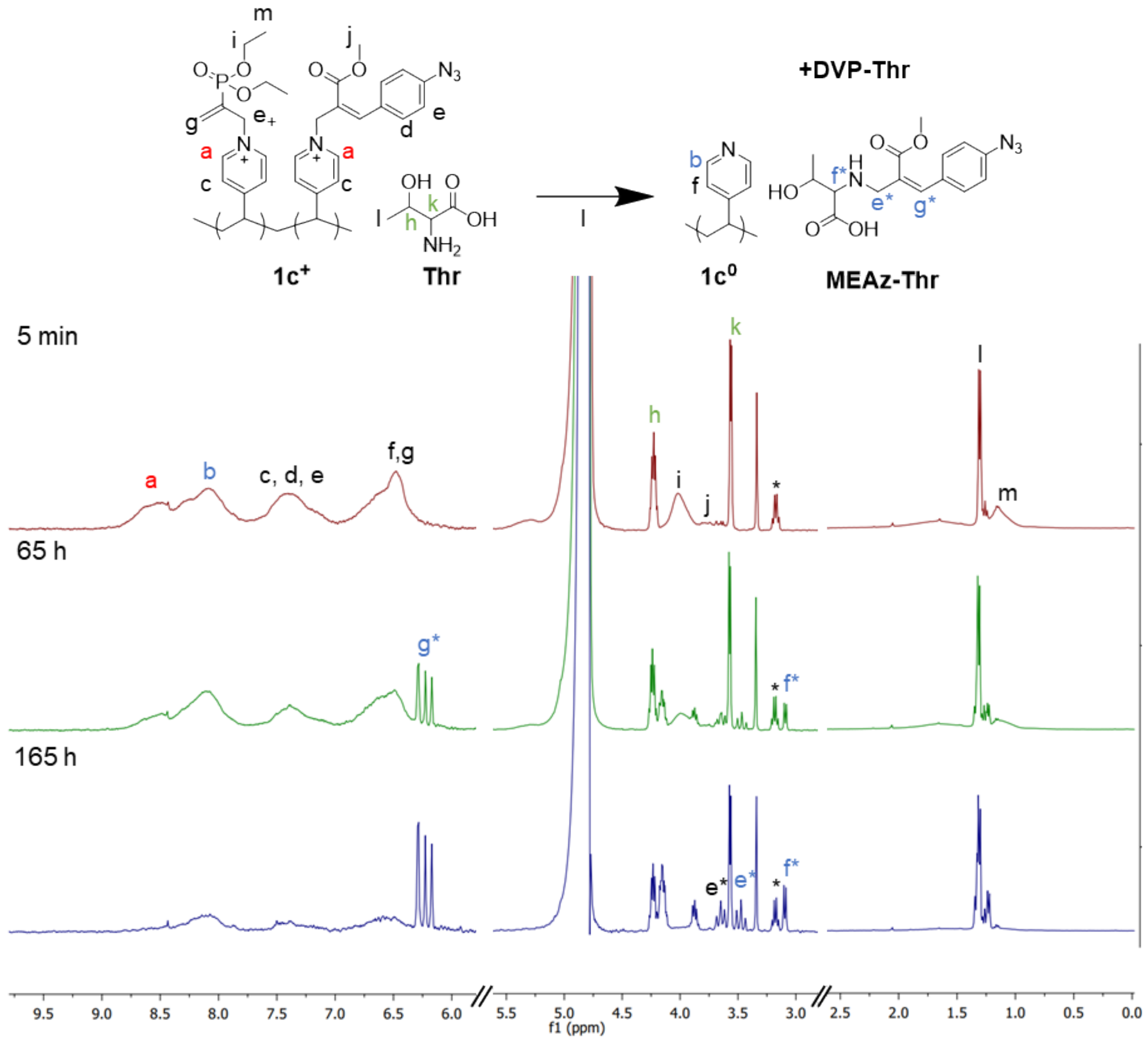

Figure S2. Example ^{1}H NMR (D_2O) spectra from reaction of cationic units on **1a** (**1a**$^+$) with glutathione (**GSH**) to form the neutralised units on **1a** (**1a**^{0}) and side product **DVP-SH**. Spectra split into 3 differently scaled sections for clarity. Integrals were standardised to an ethanol internal standard (*), with cationic conversion evaluated based on red (**1a**$^+$), and blue (**DVP-GSH**) signals. See **Figure 1** for kinetic trace.

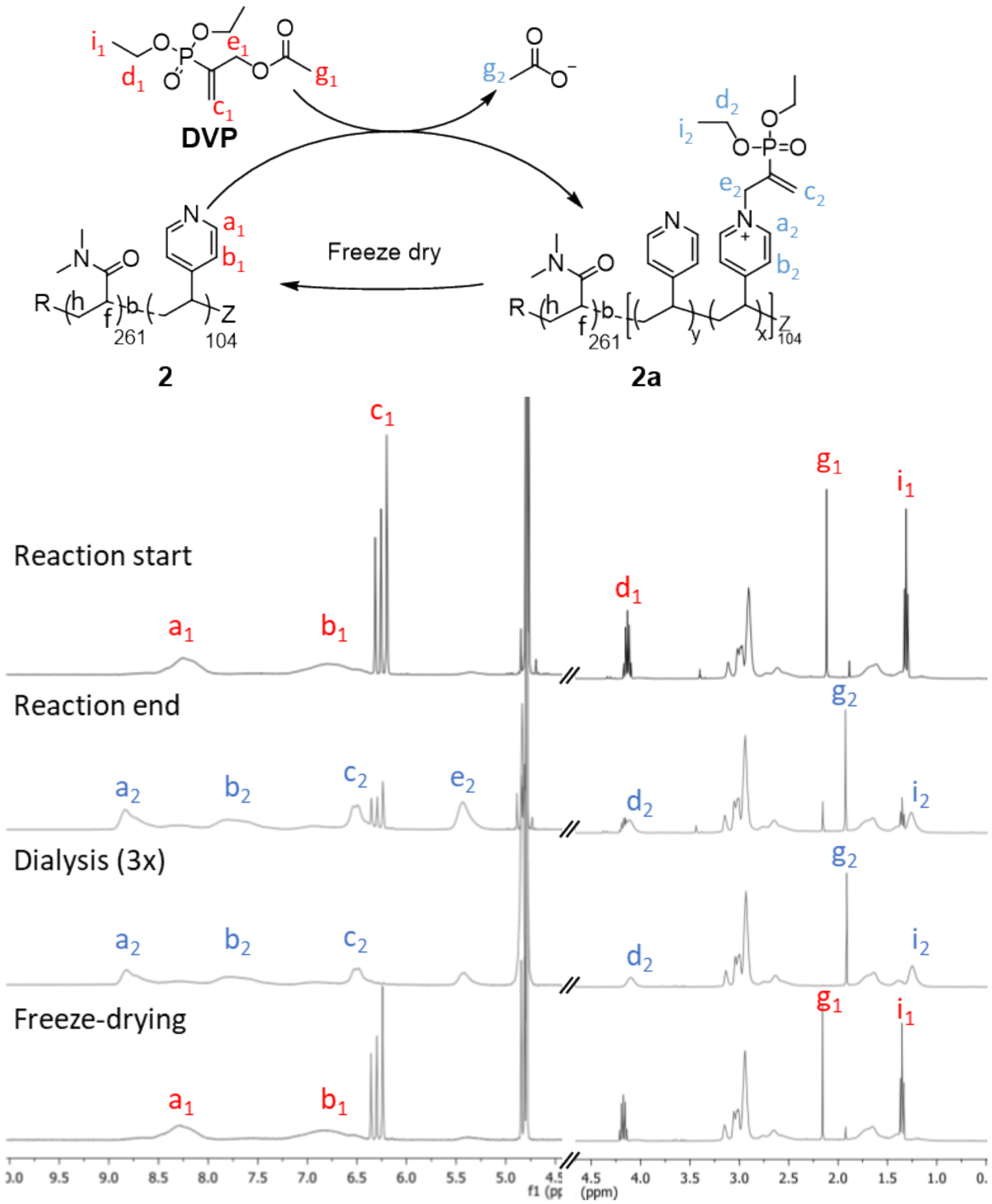

Figure S3. Example ^{1}H NMR (D_2O) for demonstration of de-cationization after freeze-drying for responsive polycation when acetate counterion is not exchanged. Specifically, 17.6 mg **2** was dissolved in 1.0 mL D_2O with 11.8 mg DVP (reaction start). After 100 h (reaction end), dialysed against DI water (3x 30 mL) (Dialysis 3x). Freeze-dried and observed ionization reduced from 66% to 27%, with a corresponding amount of DVP regenerated (Freeze-drying). Exchanging the acetate counterion with a chloride (dialysis with NaCl) allows for isolation through freeze-drying without loss in ionization.

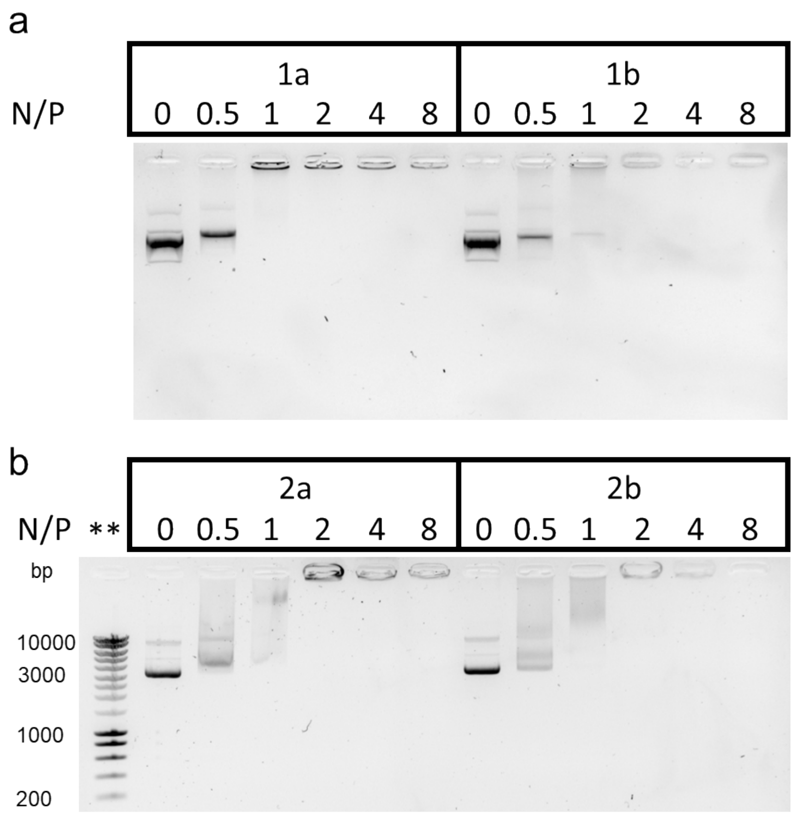

Figure S4. Gel electrophoresis data for polycation pDNA titration study. a) Increasing amounts of polycations **1a**, **1b** (a) and **2a, 2b** (b) were added to pDNA (equating to N/P from 0.5 to 8), then analysed by gel electrophoresis. Each experiment had the same pDNA concentration (20 µg/mL), loaded 10 µL/ well for analysis. ** First well is a ladder reference (Smart ladder 200 – 10000 bp, Eurogentec).

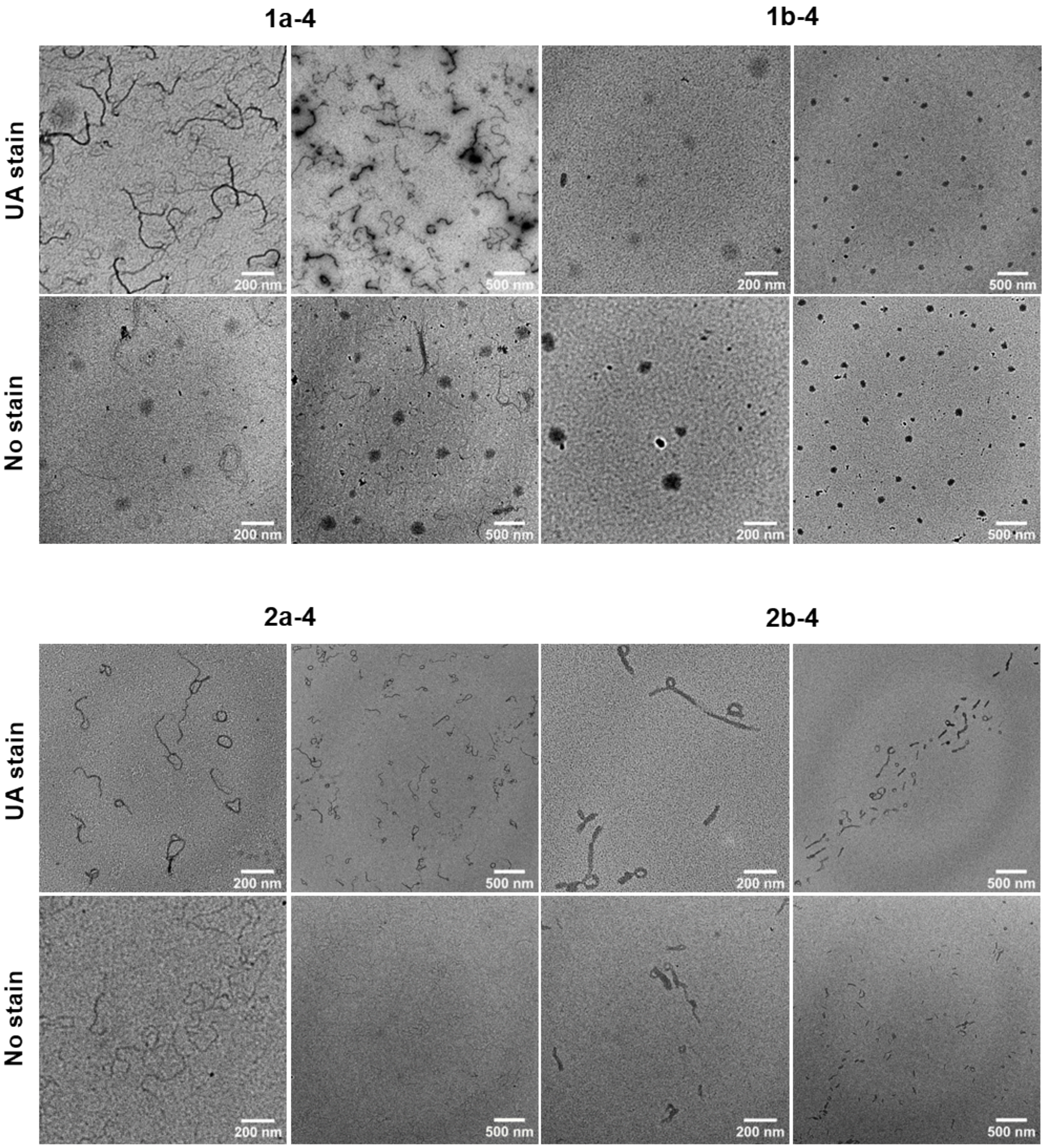

Figure S5. Additional TEM images (both stained and un-stained) for N/P = 4 polyplexes and micelles (additional data from Figure 2).

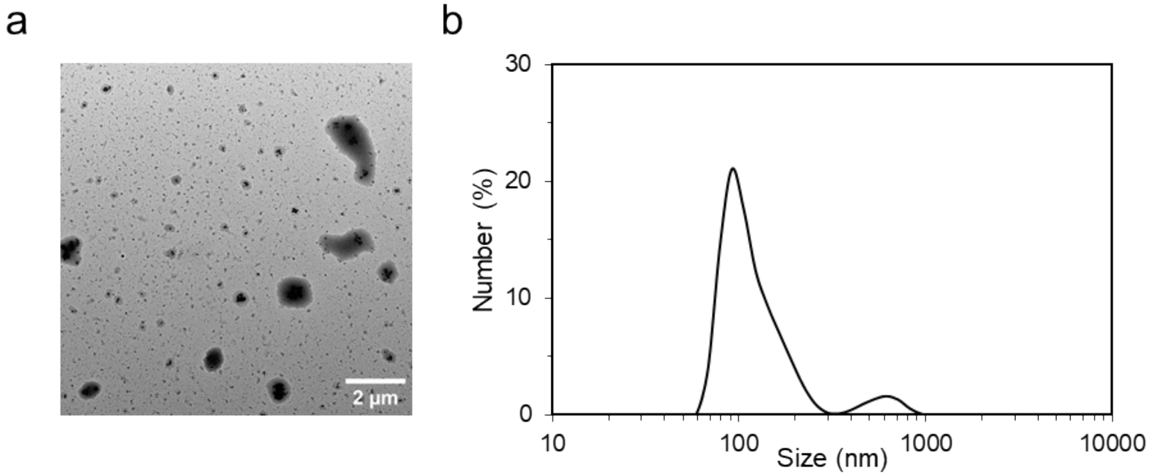

Figure S6. Demonstration of bimodal species distribution for **1b-4**. a) TEM (non-stained) of **1b-4** demonstrating species > 1 µm which were observed much less than the smaller ~100 nm species. b) Representative number intensity DLS trace for **1b-4** in 10 mM pH 7.4 PB demonstrating bimodal distribution with fraction of smaller and larger species.

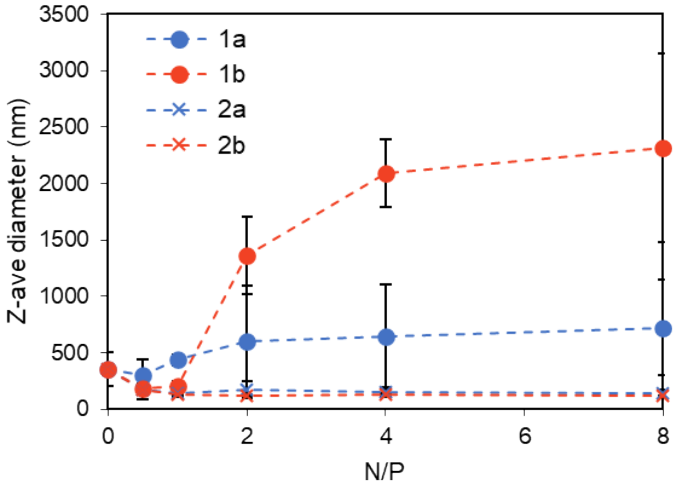

Figure S7. Change in size (Z-ave diameter) during titration of polycations to pDNA (6.5 µg/mL pDNA; 20 µM phosphates in 10 mM pH 7.4 PB) as a function of N/P (additional data from Figure 2).

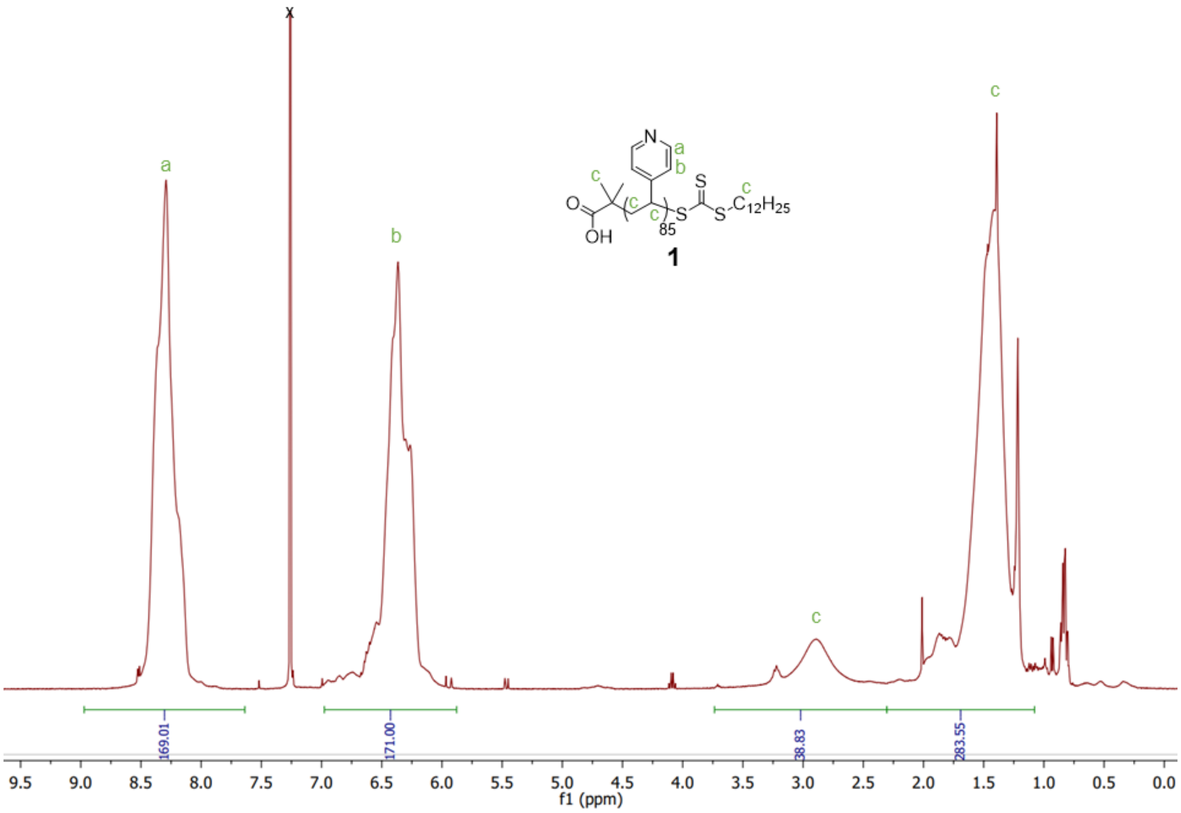

Figure S8. ^{1}H NMR ($CDCl_3$) of **1**. Polymer degree of polymerisation (DP) was determined by conversion data obtained during the polymerisation based on an ideal RAFT process ([$4VP_0$]:[CTA] = 120:1, monomer conversion = 71%, DP = 85).

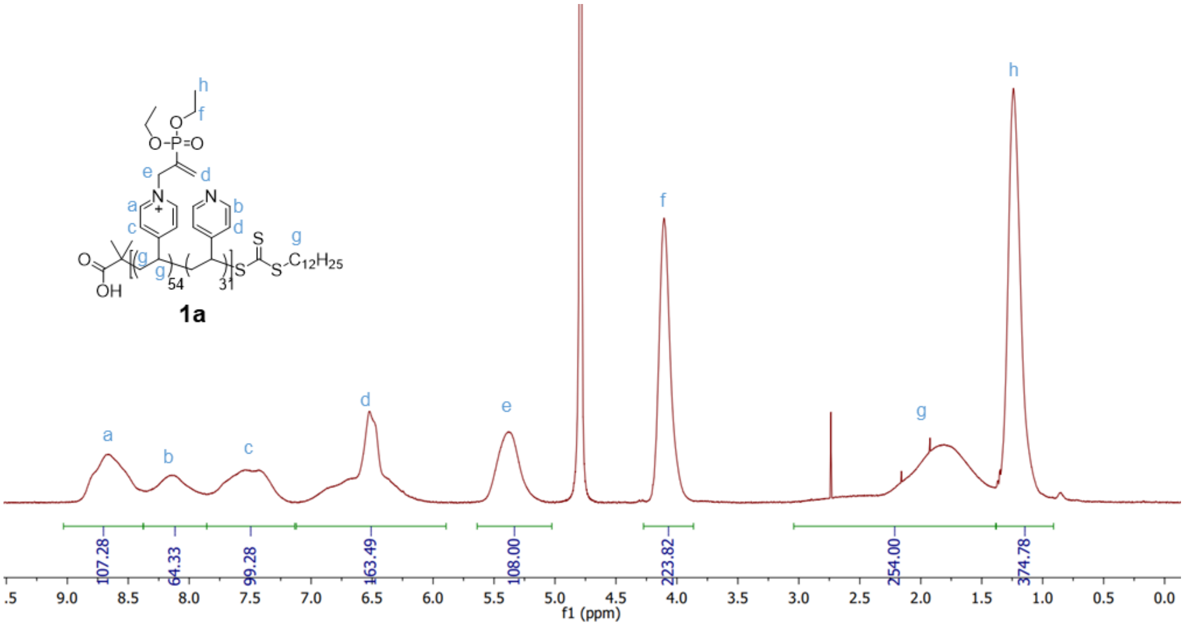

Figure S9. ^{1}H NMR (D_2O) of **1a**. Degree of PVP cationization determined by comparing ratio of neutral to cationic PVP peaks. i.e. [∫(a+c+e+f)/(2+2+2+4)]/[∫(b)/(2)] = 53.8/32.2 = 1.67. Then applying this ratio to the DP previously determined for **1** (85). PVP^+ = (85 x 1.7)/2.7 = 54 (63%).

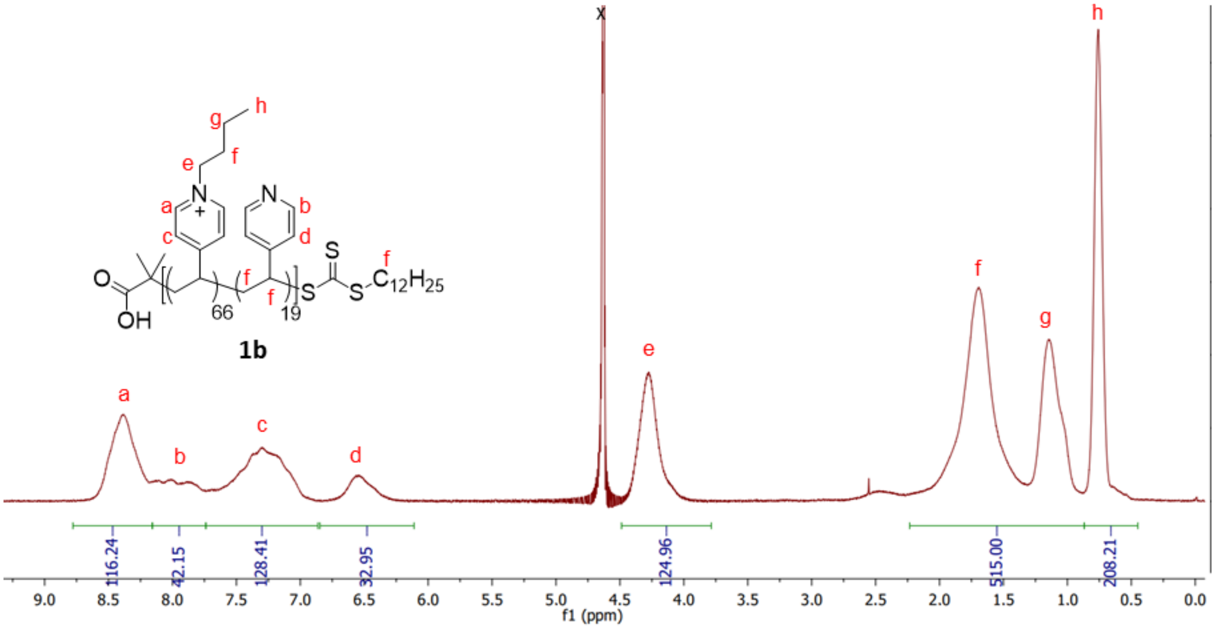

Figure S10. ^{1}H NMR (D_2O) of **1b**. Degree of PVP cationization determined by comparing ratio of neutral to cationic PVP peaks. i.e. [∫(a+c+e+h)/(2+2+2+3)]/[∫(b+d)/(2+2)] = 64.2/18.8 = 3.4. Then applying this ratio to the DP previously determined for **1** (85). PVP^+ = (85 x 3.4)/4.4 = 66 (77%).

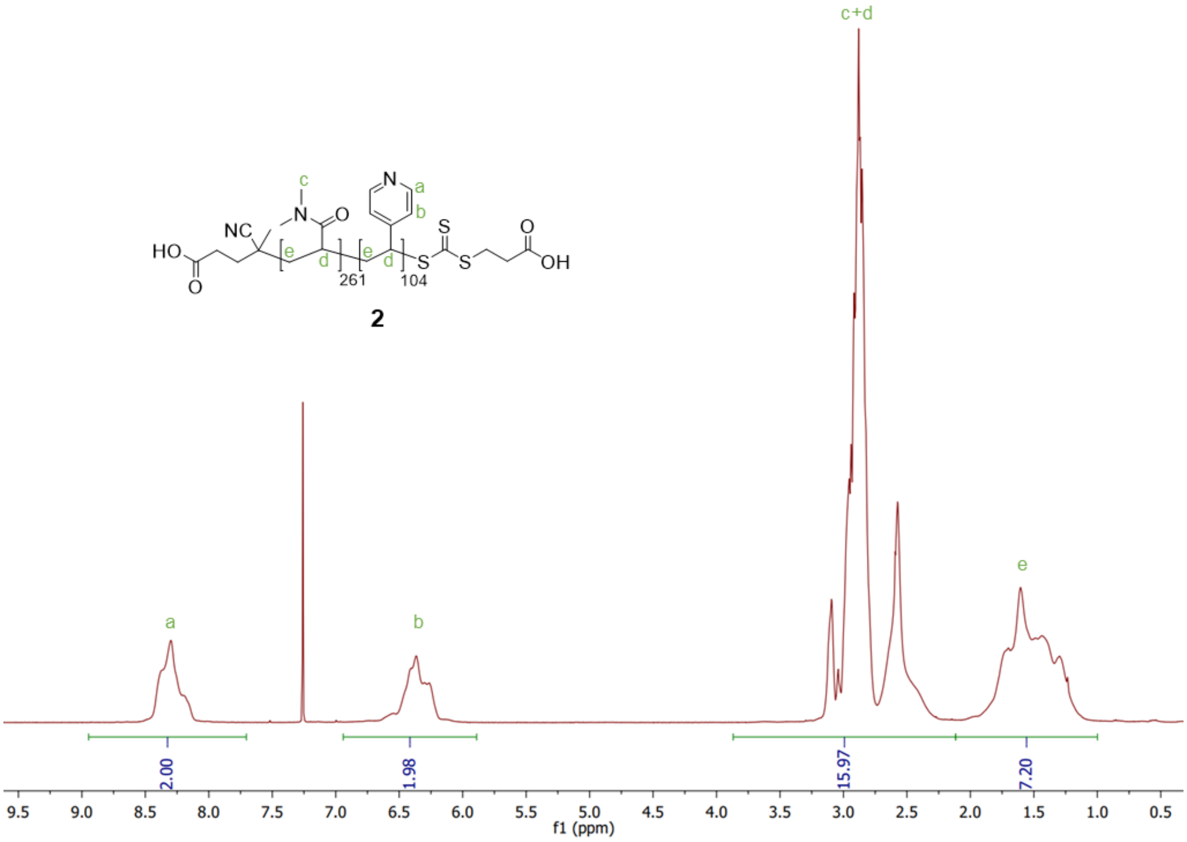

Figure S11. ^{1}H NMR (D_2O) of **2**. Polymer DP was determined by conversion data obtained during the polymerisation based on an ideal RAFT process. Ratio PVP:pDMA is ~as expected. [∫(a+b)/(2+2)]/[∫(c+d+e)-3x∫(a+b)/(4)]/(6+1+2) = 1.0/2.2 = 0.45. $Ratio_{theoertical}$ = 1 / (261/104) = 1/2.5 = 0.40.

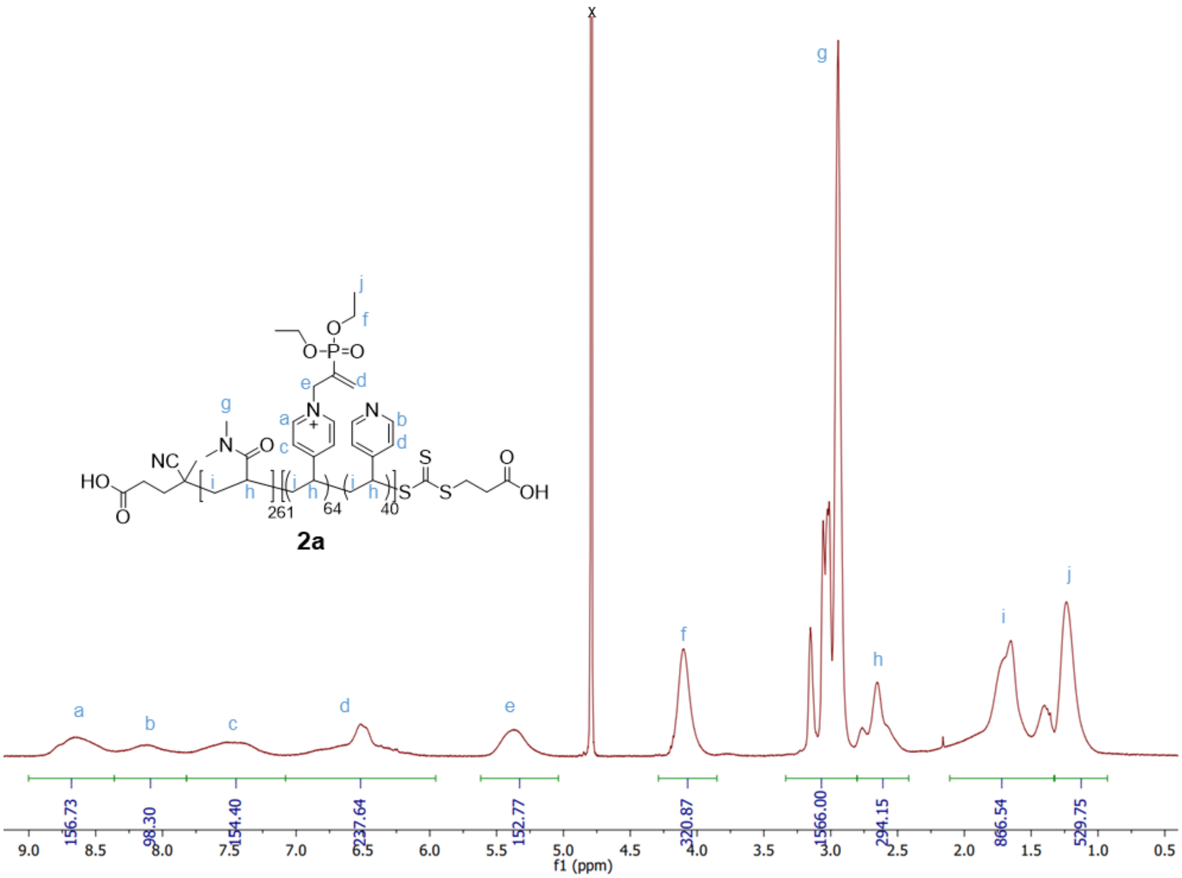

Figure S12. ^{1}H NMR (D_2O) of **2a**. Degree of PVP cationization determined by comparing ratio of neutral to cationic PVP peaks. i.e. $[\int(a+c+e+f+j)/(2+2+2+4+6)]/[\int(b)/(2)] = 82.2/49 = 1.7$. Then applying this ratio to the DP previously determined for the PVP block (104). $PVP^{+} = (104 \times 1.7)/2.7 = 64$ (61%).

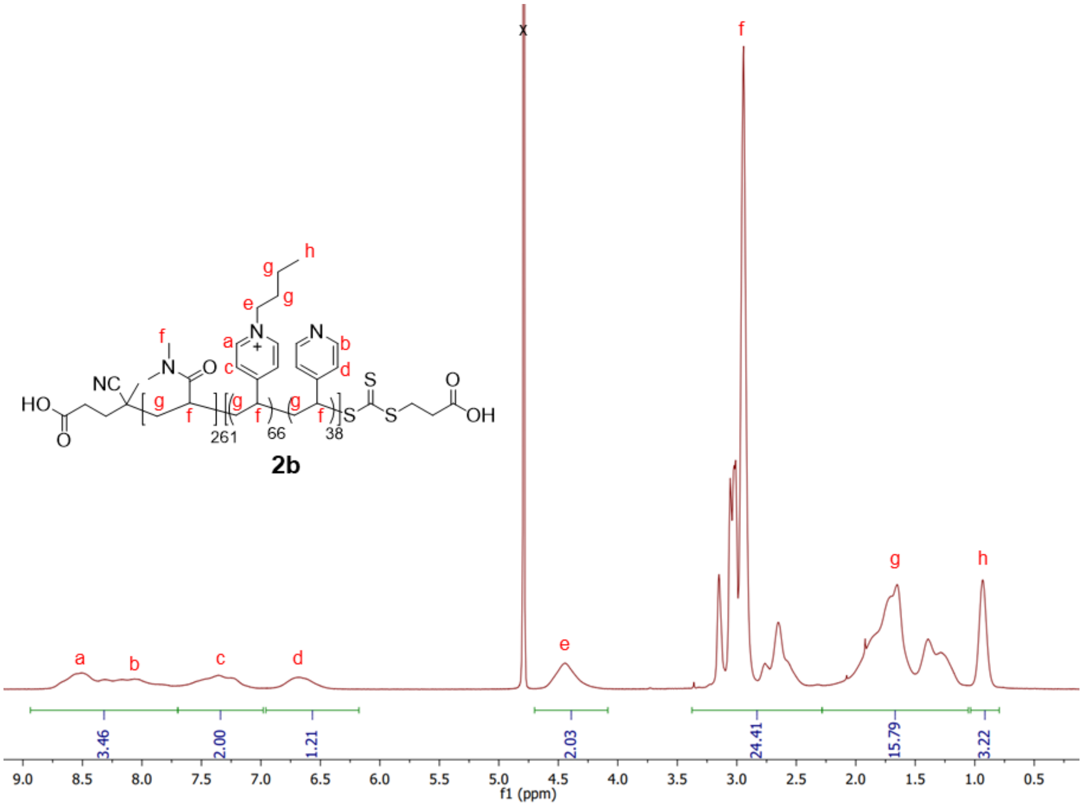

Figure S13. ^{1}H NMR (D_2O) of **2b**. Degree of PVP cationization determined by comparing ratio of neutral to cationic PVP peaks. i.e. [∫(c+e+h)/(2+2+3)]/[∫(d)/(2)]=1.04/0.61 = 1.72. Then applying this ratio to the DP previously determined for the PVP block (104). PVP^{+} = (104 x 1.7)/2.7 = 66 (63%).

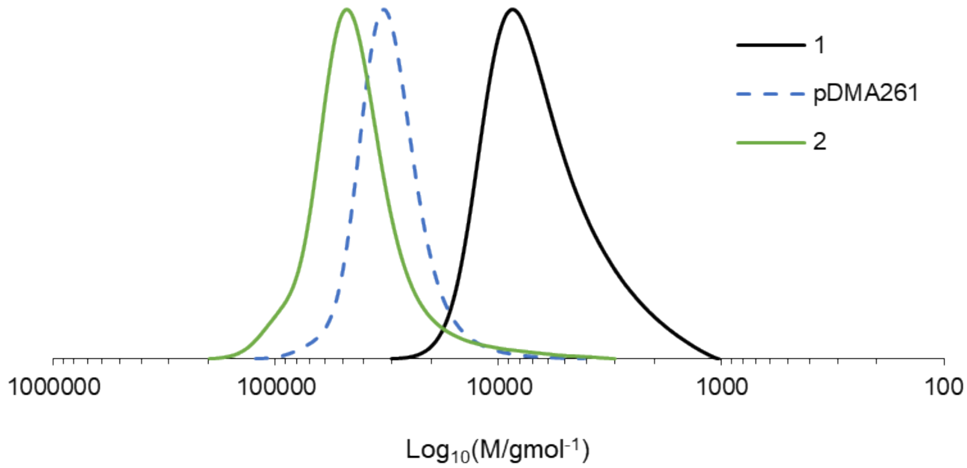

Figure S14. GPC Chromatograms (DMF, LiBr 25 mM) for selected polymers. Note shift in GPC trace from **pDMA$_{261}$** to **2**, indicating successful chain extension.

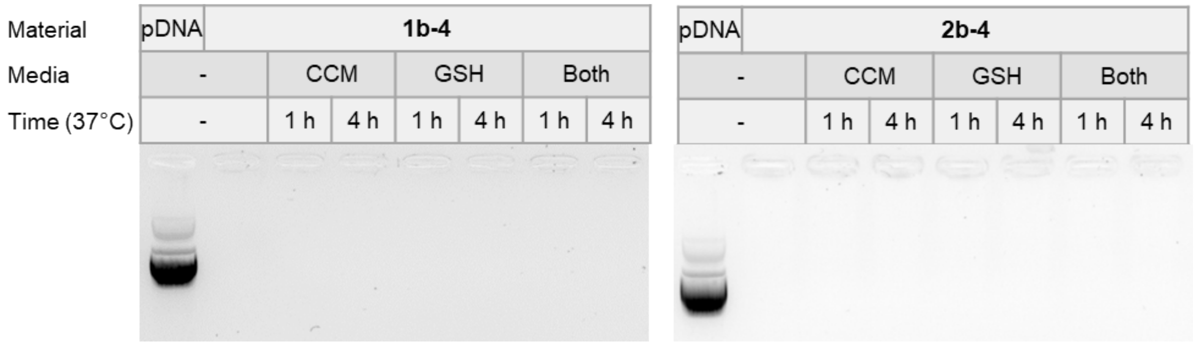

Figure S15. Analysis of pDNA release by gel electrophoresis for control complexes **1b-4** and **2b-4**. Experiment demonstrates no release in all conditions studied, as expected for the non-responsive complexes. Data relates to Figure 3.

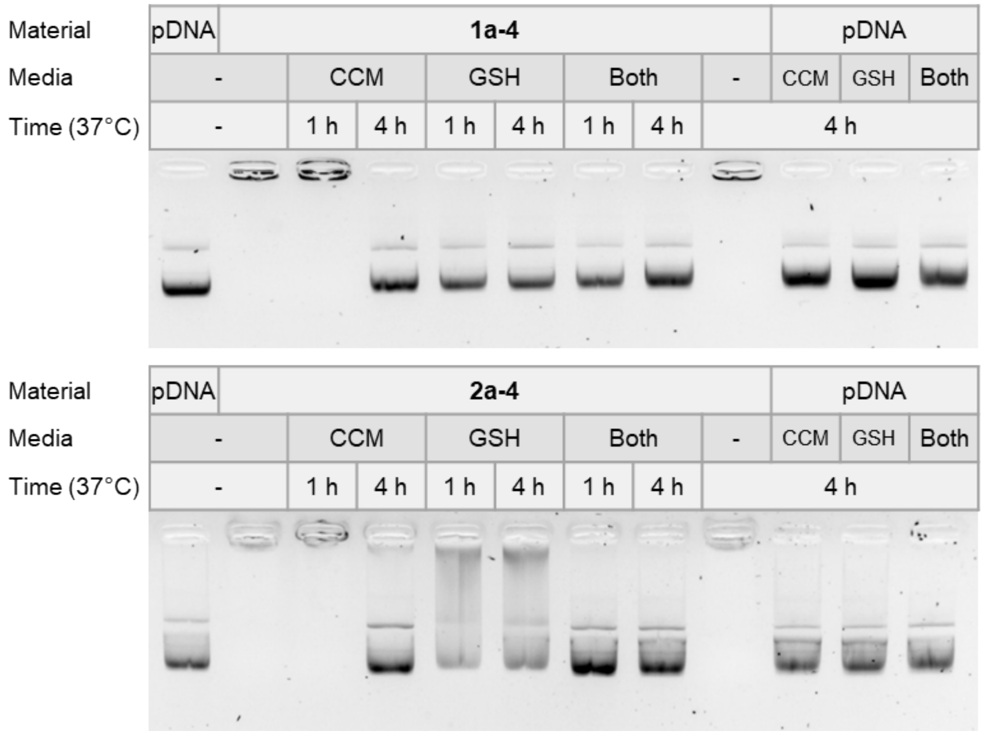

Figure S16. Additional control data (last 4 wells) for pDNA release by gel electrophoresis for responsive complexes **1a-4** and **2a-4**. Experiment demonstrates no release in 10 mM PB (-) after 4 h at 37°C, while pDNA is not affected by 4 h incubation in cell culture media (CCM), 1 mM glutathione (GSH) or both combined. Data relates to Figure 3. Note: presence of two bands in pDNA only lane due to supercoiled and open circle forms.

Table S2. Effect of polymer **2** (product after decationization of **2a**) on the zeta potential of pDNA in cell culture media (CCM) and 1 mM glutathione (GSH). Data relates to Figure 3.

Species	ZP (CCM), mV	ZP (GSH), mV
pDNA	-16.9 ± 1.4	-23.5 ± 1.2
pDNA + 2 (N/P = 4)	-11.6 ± 1.3	-13.3 ± 1.1
pDNA + 2 (N/P = 16)	-6.3 ± 0.8	-5.8 ± 0.4

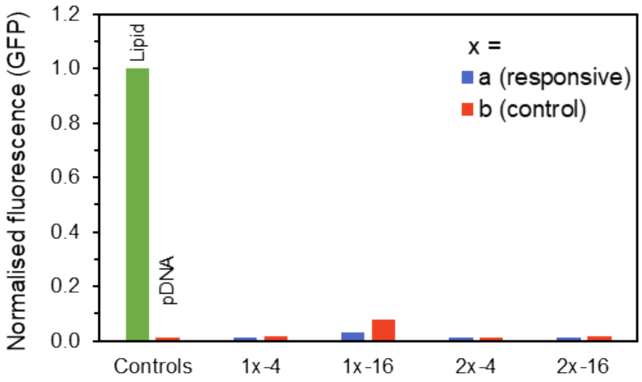

Figure S17. Normalized GFP fluorescence intensity of CHO cells 48 h after treatment with pDNA (1 µg/well) micelles and polyplexes as measured by CLSM (λ_{ex} = 458 nm, detection range λ_{det} = 493 – 797 nm). The results are compared to a lipid formulation (DOPE/DC-Chol:70/30). Example images from study are shown in Figure S18.

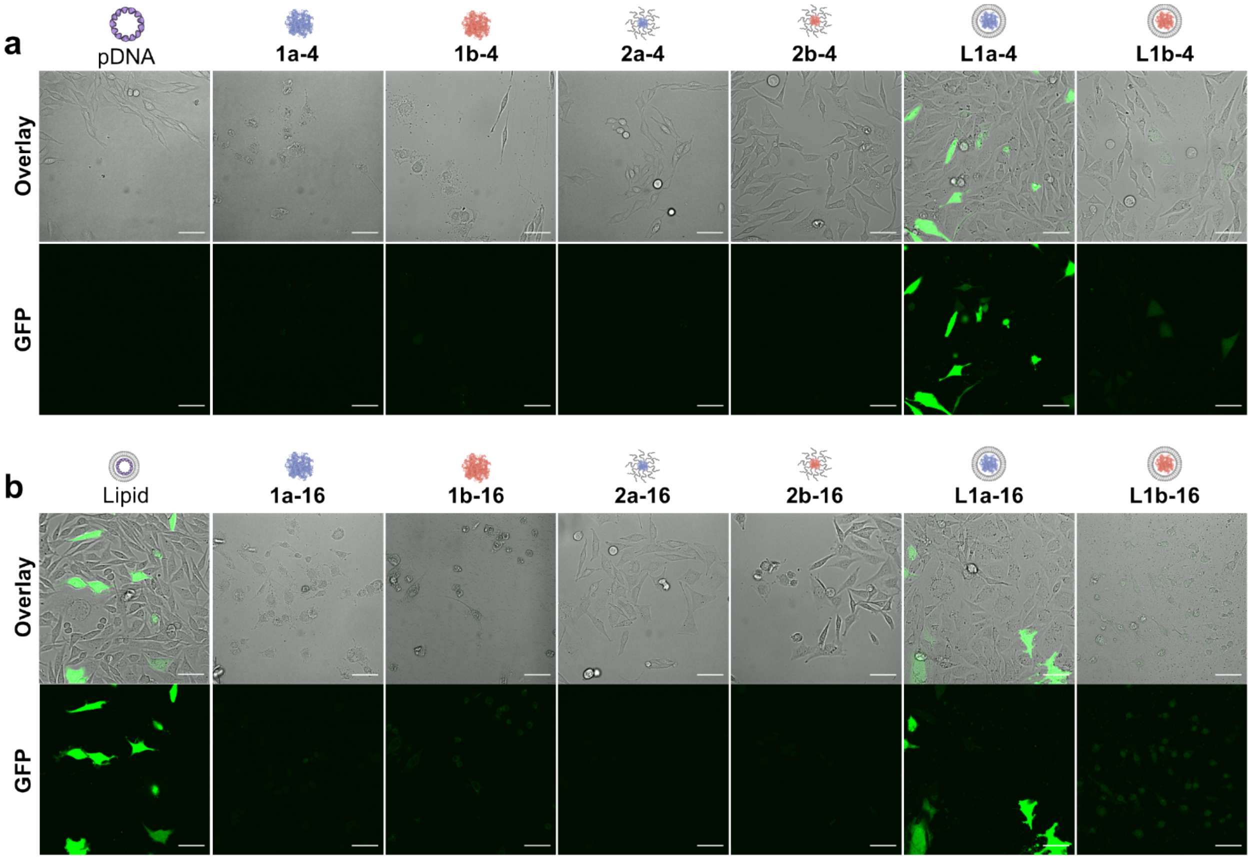

Figure S18. CLSM images of CHO cells 48 h after treatment with various pDNA complexes (1 µg pDNA / well), studying extent of EGFP transfection. a) pDNA only and N/P = 4 complexes, b) Lipid pDNA complex (Lipid) and N/P = 16 complexes. Images are processed to have matching contrast in GFP channel. Scale bar is 50 µm. Note polyplex treated cells have signs of cell death (cells low in number, rounded up and shrunken) and observed faint GFP signal may be an autofluorescence response. Data relates to Figure 4b.

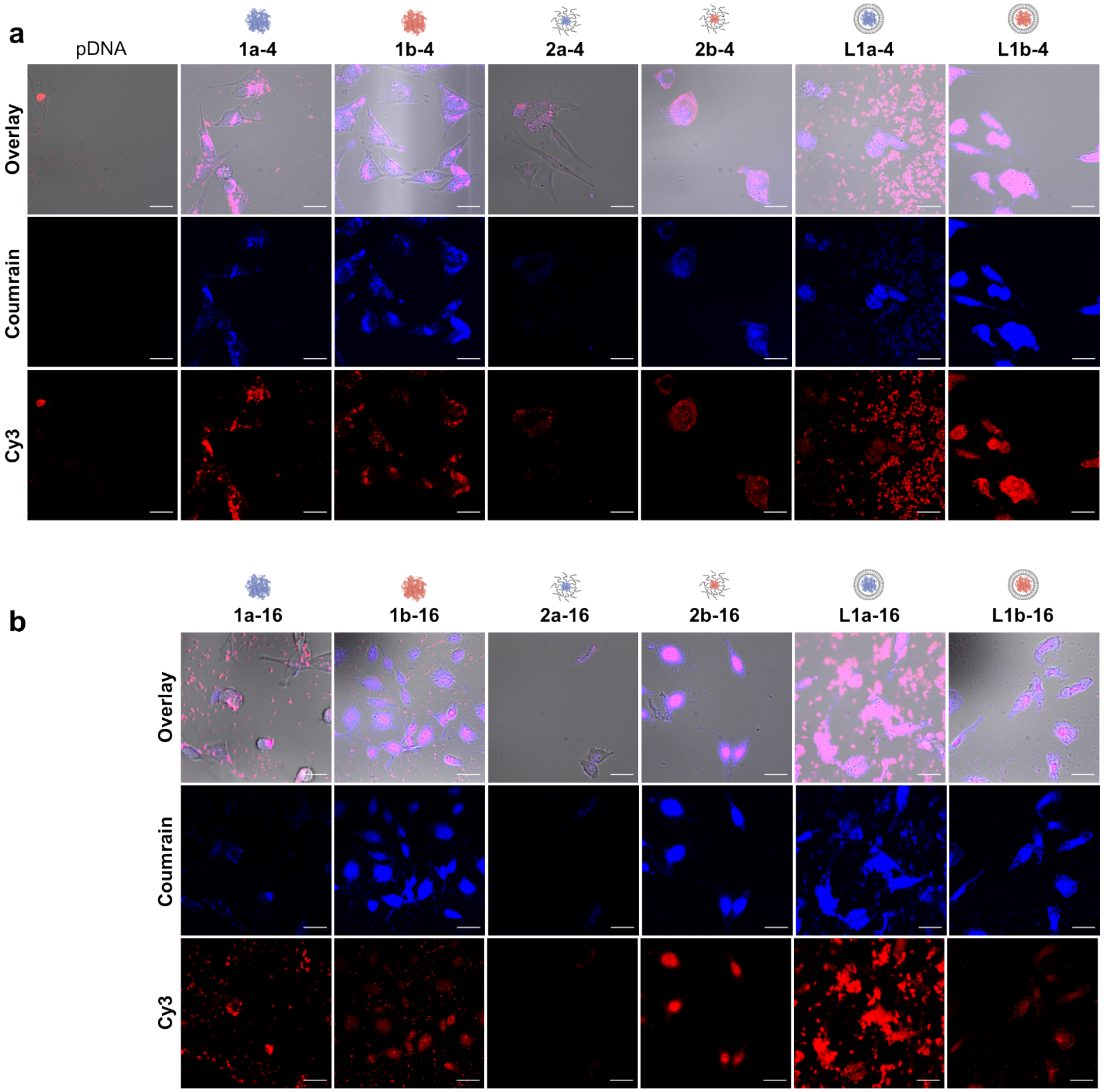

Figure S19. CLSM images of CHO cells 4 h after treatment (washed 3 times with cell culture media) with various fluorescently labelled pDNA complexes (1 μg pDNA / well). Extent of cellular uptake of polymer (blue, Coumarin labelled) and pDNA (red, Cy3 labelled) is analysed. a) pDNA only and N/P = 4 complexes, b) N/P = 16 complexes. Images are processed to have matching contrast in each fluorescence channel. Scale bar is 25 μm. Data relates to Figure 4b.

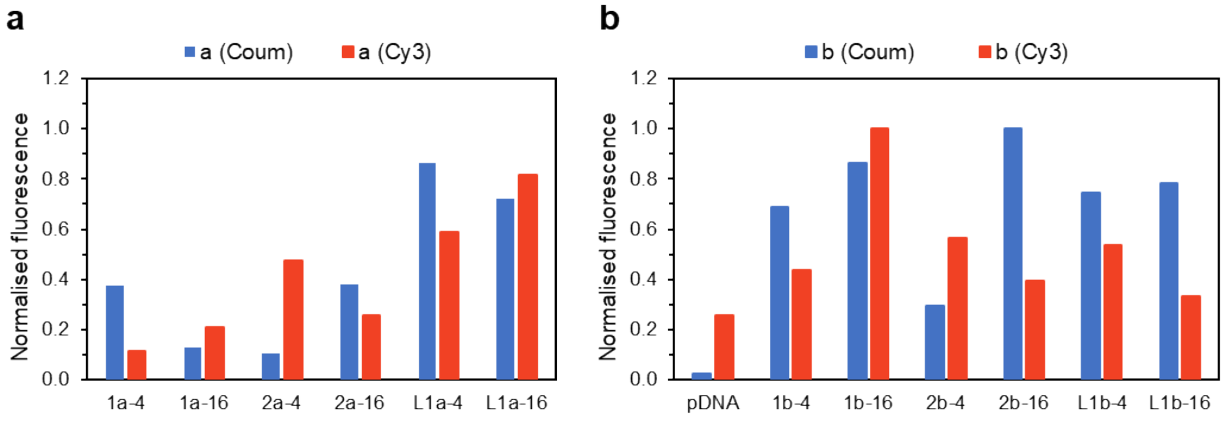

Figure S20. Normalised fluorescence intensity quantitated from CLSM images of CHO cells 4 h after treatment (washed 3 times with cell culture media) with various fluorescently labelled pDNA complexes (1 μg pDNA / well). Extent of cellular uptake of polymer (blue, Coumarin labelled) and pDNA (red, Cy3 labelled) is analysed. a) responsive complexes, b) control complexes. Data relates to Figure 4b.

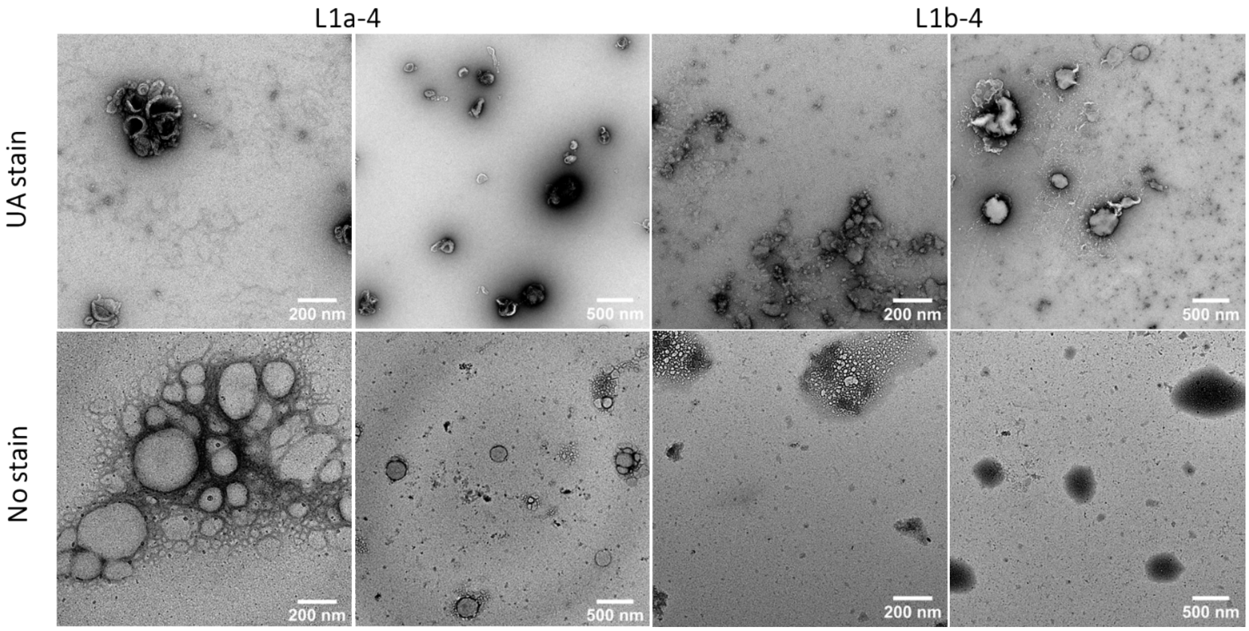

Figure S21. Additional TEM images (both stained and un-stained) for N/P = 4 lipopolyplexes. Data relates to Figure 4b. Layered or walled structure evident in UA-stained images for both complexes.

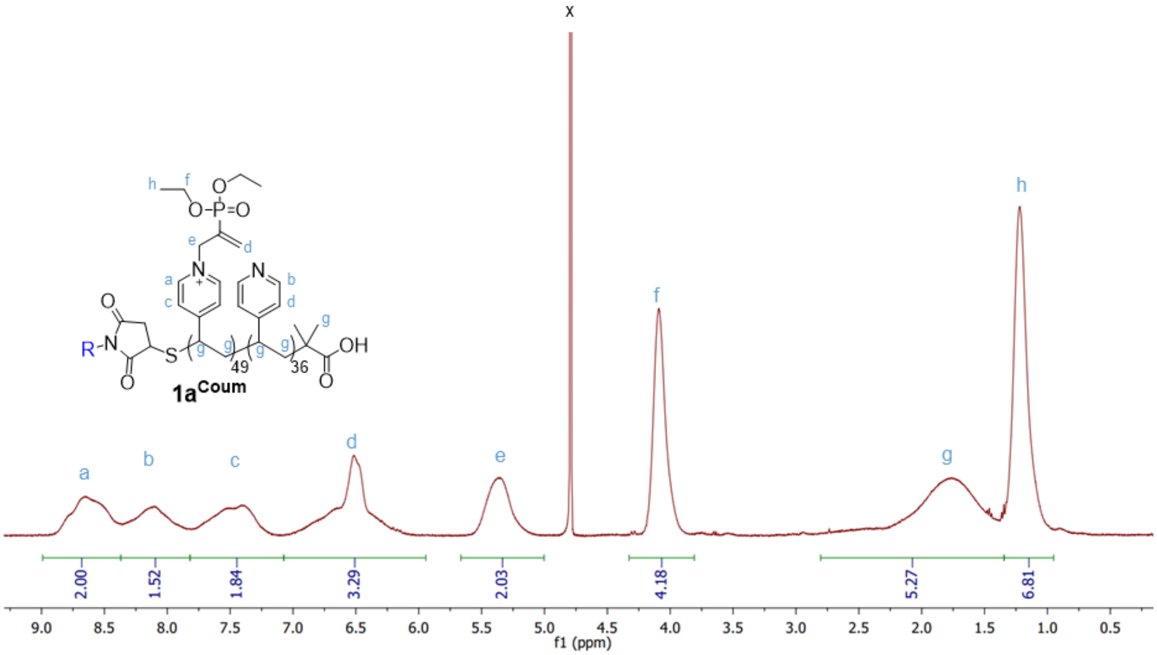

Figure S22. ^{1}H NMR (D_2O) of **1a$^{\mathbf{Coum}}$**. Degree of PVP cationization determined by comparing ratio of neutral to cationic PVP peaks. i.e. $[\int(a+c+e+f+h)/(2+2+2+4+6)]/[\int(b)/(2)] = 1.05/0.76 = 1.39$. Then applying this ratio to the DP previously determined for the PVP block (85). $PVP^{+} = (85x\ 1.4)/2.4 = 49$ (58%). R is coumarin moiety, see **Scheme S1** for structure.

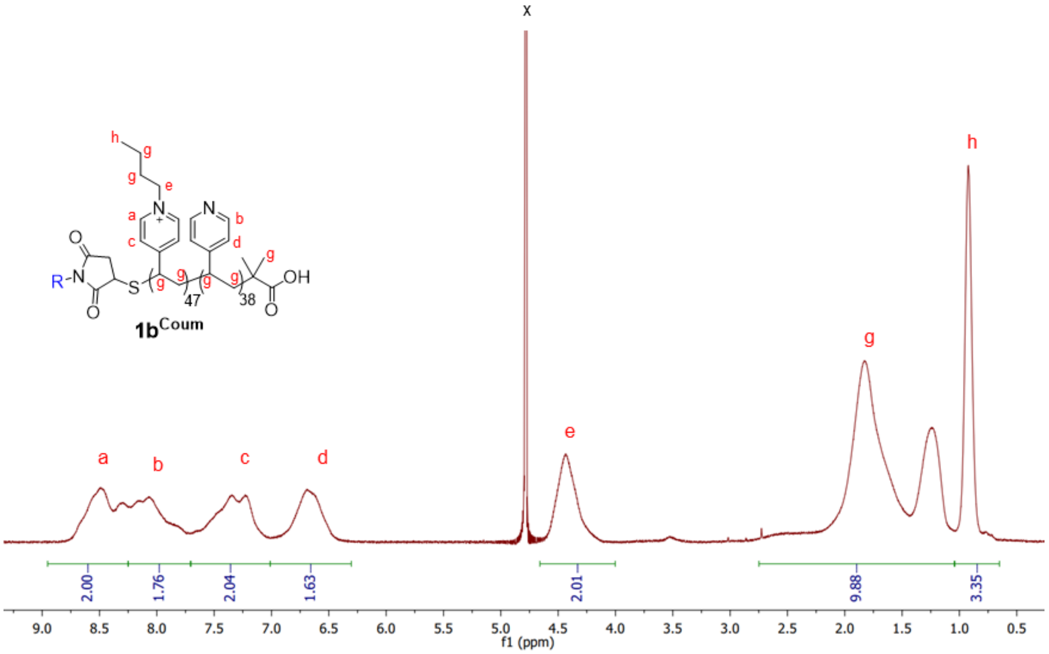

Figure S23. ^{1}H NMR (D_2O) of **1b$^{\mathbf{Coum}}$**. Degree of PVP cationization determined by comparing ratio of neutral to cationic PVP peaks. i.e. [∫(a+c+e+h)/(2+2+2+3)]/[∫(b+d)/(2+2)] = 1.04/0.85 = 1.2. Then applying this ratio to the DP previously determined for the PVP block (85). PVP$^+$ = (85 x 1.2)/2.2 = 47 (55%). R is coumarin moiety, see **Scheme S1** for structure.

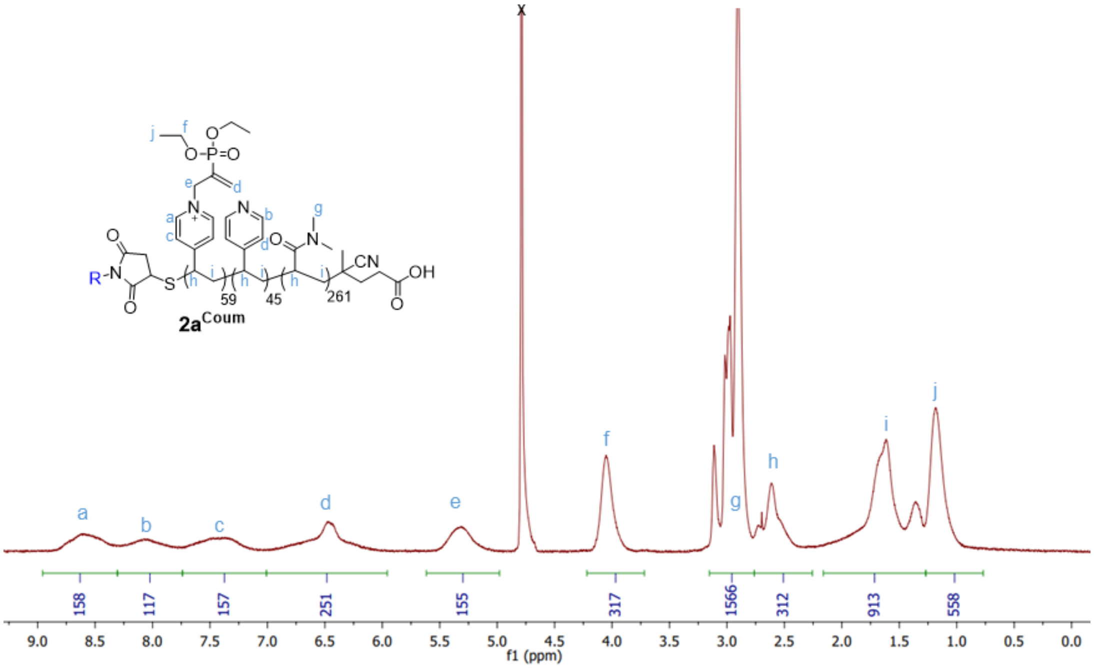

Figure S24. ^{1}H NMR (D_2O) of **2a^{Coum}**. Degree of PVP cationization determined by comparing ratio of neutral to cationic PVP peaks. i.e. [∫(a+c+e+f)/(2+2+2+4)]/[∫(b)/(2)] = 78.7/58.5 = 1.35. Then applying this ratio to the DP previously determined for the PVP block (104). PVP$^+$ = (85x 1.35)/2.35 = 59 (57%). R is coumarin moiety, see **Scheme S1** for structure.

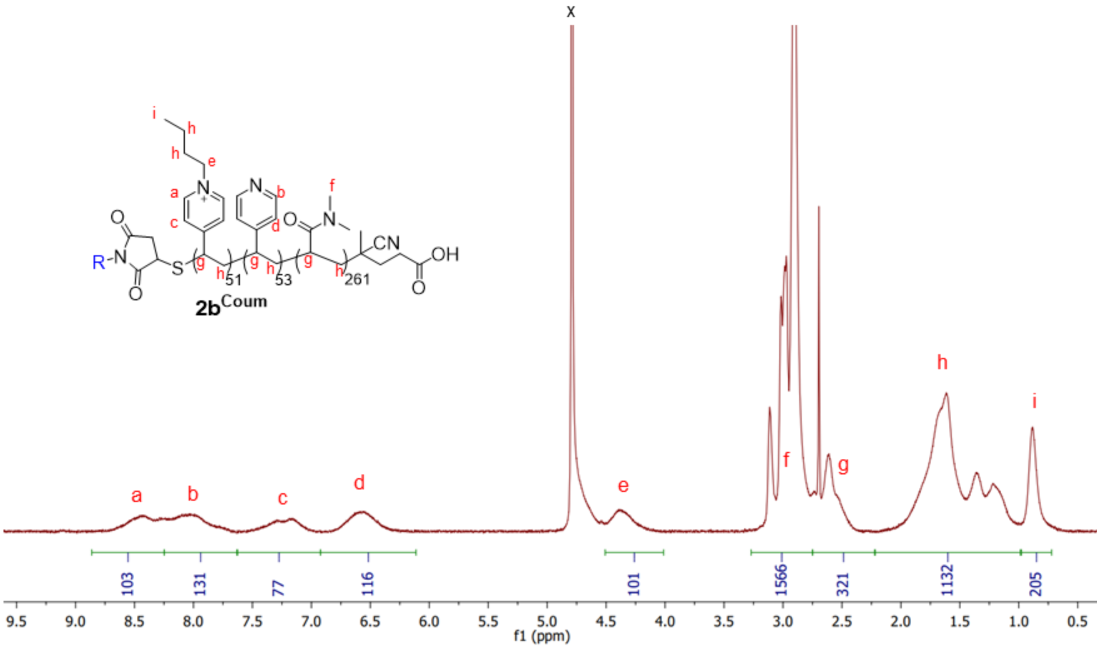

Figure S25. ^{1}H NMR (D_2O) of **2b^{Coum}**. Degree of PVP cationization determined by comparing ratio of neutral to cationic PVP peaks. i.e. [∫(a+c+e+h)/(2+2+2+3)]/[∫(b+d)/(2+2)] = 54/57 = 0.94. Then applying this ratio to the DP previously determined for the PVP block (104). PVP$^+$ = (104 x 0.94)/1.94 = 51 (49%). R is coumarin moiety, see **Scheme S1** for structure.

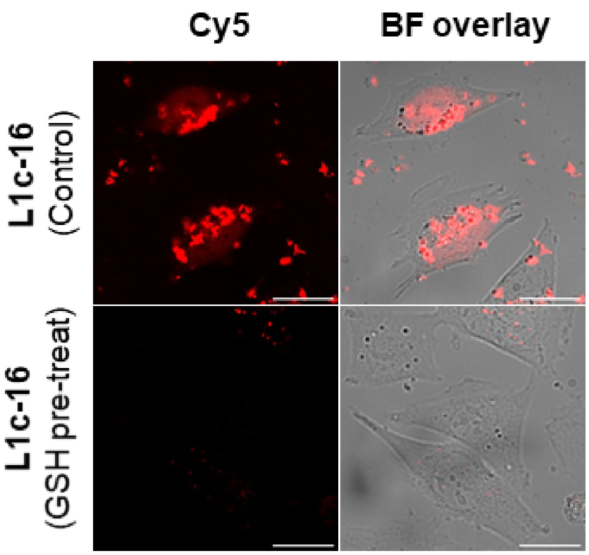

Figure S26. Cy5 (red) localisation study of CHO cells treated with lipopolyplexes prepared using **1c** (**L1c-16,** Control). These are compared to cells treated with matching lipopolyplexes prepared from **1c** pre-treated with 5x excess GSH (**L1c-16,** GSH pre-treat). Images were taken using CLSM with Cy5 (λ_{ex} = 633 nm, λ_{det} = 638 – 797 nm, red) and bright field channels overlayed, scale bar is 25 µm. Data relates to Figure 5c.

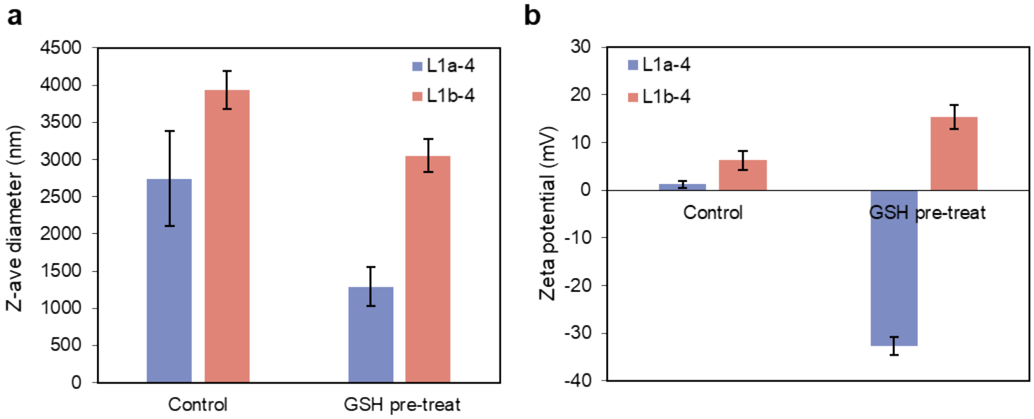

Figure S27. Change in size and surface charge of lipopolyplexes after GSH pre-treatment used in Figure 5b – 5d to induce 'extracellular deionization' for the responsive polycations (n = 2). Complexes were prepared as standard then diluted into CCM (phenol red free) for measurement of 'control' state. Then GSH was added (to 2 mM) and the complexes were incubated at 37°C for 1 h before a second measurement (GSH-pre-treat). Note: the larger initial Z-ave sizes for the control complexes is likely due to measurement in cell culture media (CCM) compared to previous data collected in 10 mM PB.

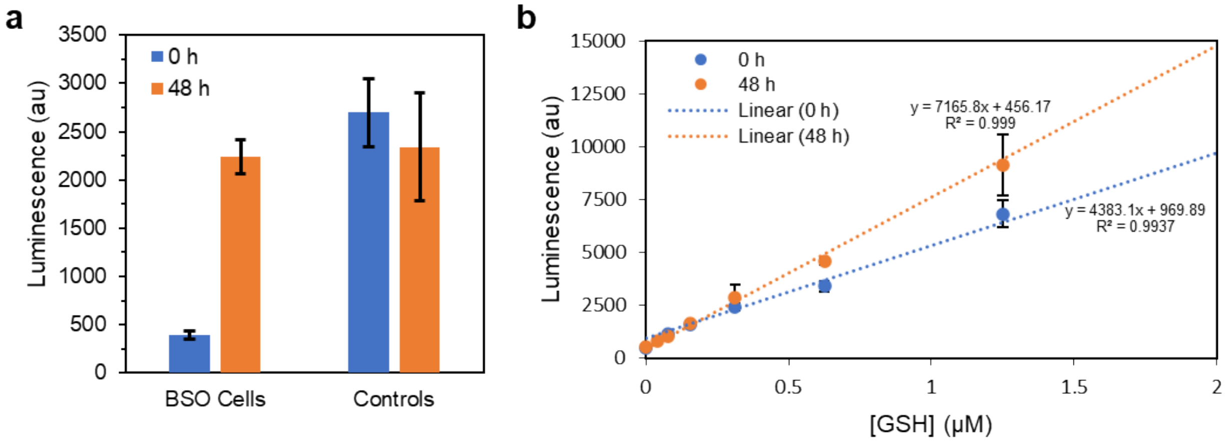

Figure S28. Additional data from intracellular glutathione study quantitated using GSH-Glo^{TM} Glutathione Assay (Promega). a) Raw luminescence data (average of 4 wells, 5 k cells / well). b) Glutathione standard curve as measured on both t = 0 and t = 48 h. Data relates to Figure 5e.

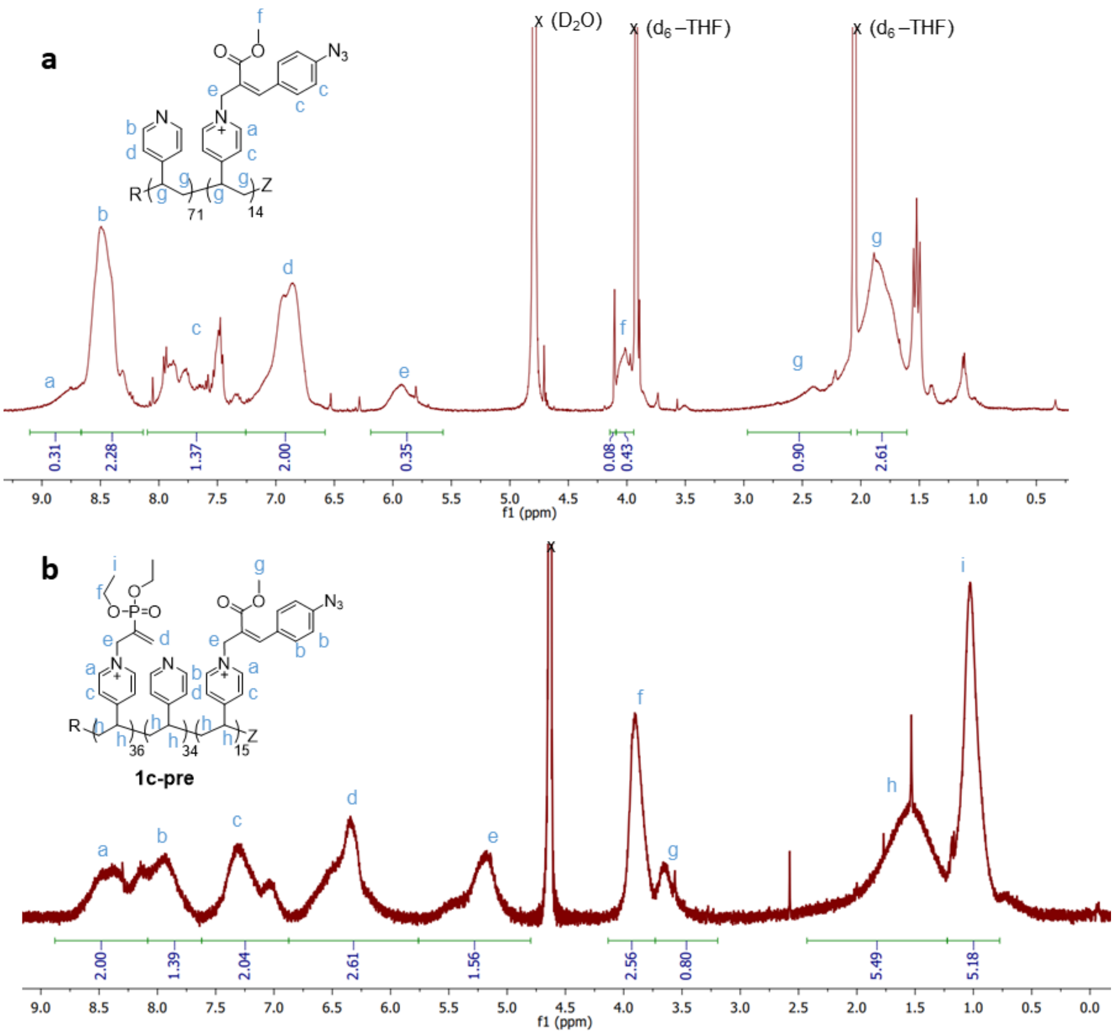

Figure S29. a) ^{1}H NMR (d_6-THF/D_2O) demonstrating crude product 4 h after addition of **ME-Az** to **1** (residue unreacted ME-Az still visible as sharp peaks showing on top of broad peaks 7.5 - 8 ppm and 4.2 ppm). b) ^{1}H NMR (D_2O) of **1c-pre.** Degree of PVP cationization determined by comparing ratio of neutral to cationic PVP peaks. i.e. $PVP^{ME\text{-}Az+}$:PVP^{DVP+}:PVP = ∫(f)/(4):∫(g)/(3):[[∫(b)-∫(g)/3]+[∫(d)-2x∫(f)/4]]/(2+2) = 0.64:0.26:0.62. Then applying this ratio to the DP previously determined for the PVP block (85). $PVP^{ME\text{-}Az+}$:PVP^{DVP+}:PVP = 36:34:15. Data relates to Figure 5a.

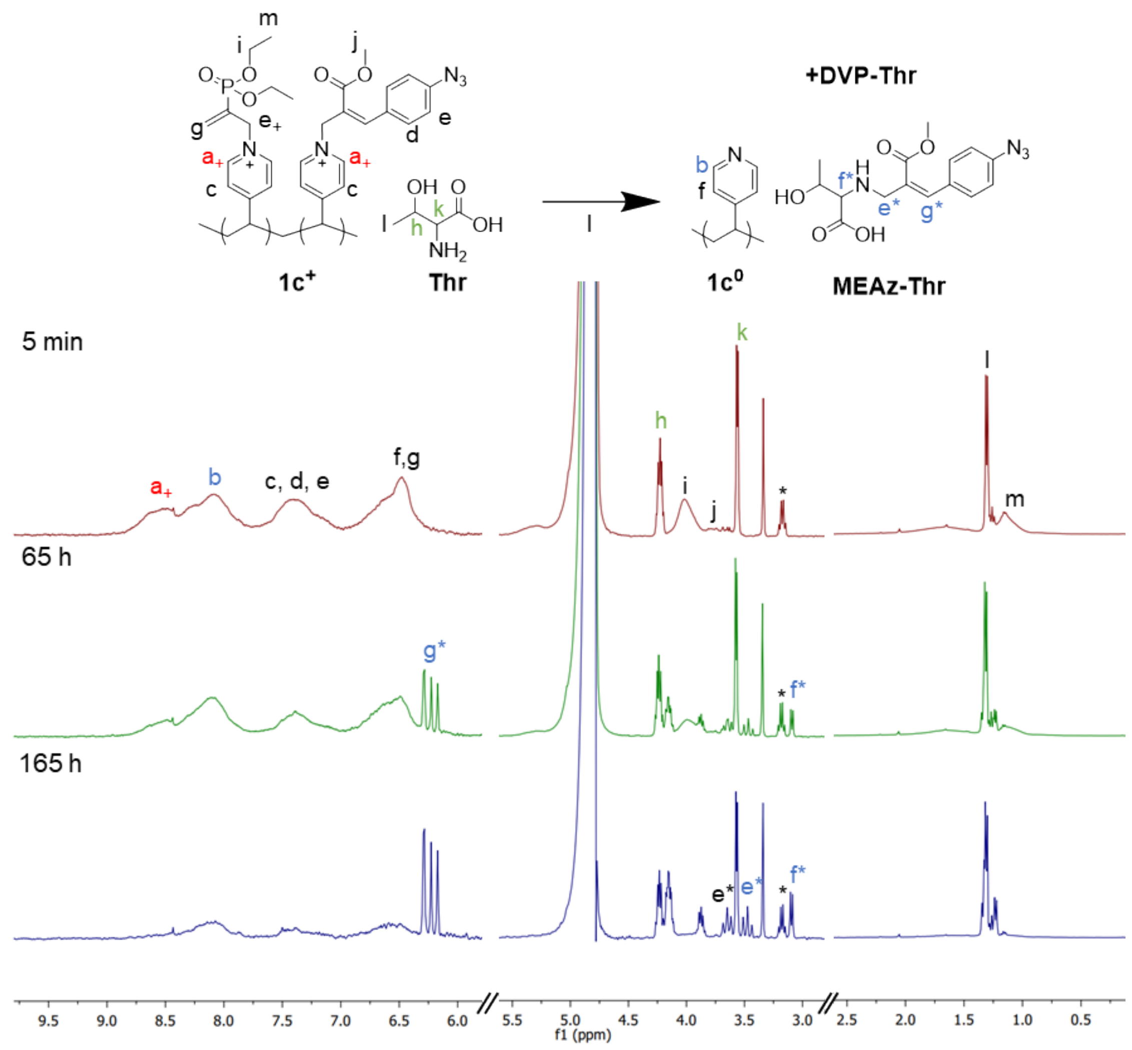

Figure S30. Example ^{1}H NMR (D_2O) spectra from the reaction of cationic units on **1c** (**1c**$^+$) with threonine (**Thr**) to form the neutralised units on **1a** (**1a**^{0}) and a side products **DVP-Thr** and **MEAz-Thr**. Spectra split into 3 differently scaled sections for clarity. Integrals were standardised to an ethanol internal standard (*), with cationic conversion evaluated based on red (**1c**$^+$), and blue (**1a**^{0}, **DVP-Thr** and **MEAz-Thr**) signals.

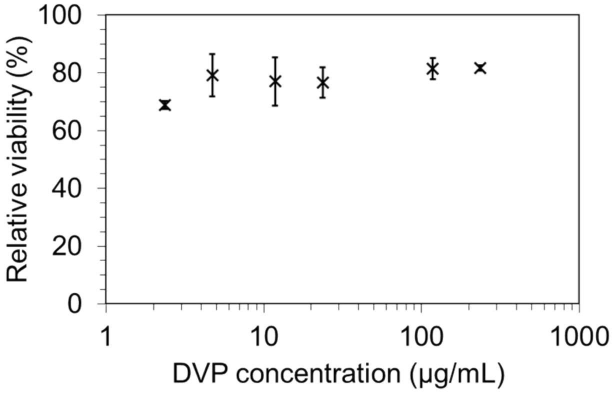

Figure S31. Cell viability of CHO cells treated with DVP (cationizing reagent used in this study) as measured via MTS assay. Data relates to Figure 6.

	1a	1b		sigmoidal four-parameter logistic model				
a	84.4	78.7		$y=a-\frac{(a-b)\times x^h}{x^h+EC50^h}$				
b	0	0						
EC50	0.467	0.055						
slope (h)	5.89	31.72						
			Model					
x (mM)	0.01	0.02	0.05	0.1	0.2	0.5	1	
1a measure	72.2	77.6	87.7	80.6	103.7	32.5	16.9	
1a model	84.37	84.37	84.37	84.36	83.81	33.75	0.94	Sum error
Error^2	147.97	46.15	11.24	14.44	395.00	1.64	253.83	870.3
1b measure	78.2	79.2	74.4	0.4	2.2	1.1	7.0	
1b model	78.70	78.70	74.38	0.00	0.00	0.00	0.00	Sum error
Error^2	0.27	0.27	0.00	0.14	4.79	1.15	49.59	56.2

Figure S32. Fitting information for viability assay on the polycations **1a** and **1b**. Fitting parameters determined by setting b = 0 (minimum viability = 0%) and minimising the squared error for a, EC_{50} and h using excels solver function. Data relates to Figure 6.

References

1. B. Klemm, R. W. Lewis, I. Piergentili and R. Eelkema, *Nature Communications*, 2022, **13**, 6242.
2. D. Széliová, D. E. Ruckerbauer, S. N. Galleguillos, L. B. Petersen, K. Natter, M. Hanscho, C. Troyer, T. Causon, H. Schoeny, H. B. Christensen, D.-Y. Lee, N. E. Lewis, G. Koellensperger, S. Hann, L. K. Nielsen, N. Borth and J. Zanghellini, *Metab. Eng.*, 2020, **61**, 288-300.
